# Mapping the Tissue‐of‐Origins of Mesenchymal Stromal Cells in Injury Repair

**DOI:** 10.1002/advs.202509533

**Published:** 2025-11-27

**Authors:** Xinyu Thomas Tang, Yiming Liam Liu, Yidi Augenstern Du, Shan−Shan Wang, Bo O. Zhou

**Affiliations:** ^1^ Key Laboratory of Multi‐Cell Systems Shanghai Institute of Biochemistry and Cell Biology Center for Excellence in Molecular Cell Science Chinese Academy of Sciences University of Chinese Academy of Sciences Shanghai 200031 China; ^2^ Department of Hepatic Oncology Liver Cancer Institute Zhongshan Hospital Fudan University and Key Laboratory of Carcinogenesis and Cancer Invasion Ministry of Education Shanghai 200032 China; ^3^ Shanghai Academy of Natural Sciences (SANS) Shanghai 200031 China

**Keywords:** cancer associated fibroblast, fibrosis, inflammation, mesenchymal stromal cells, myofibroblast

## Abstract

Culture‐expanded mesenchymal stromal cells (MSCs) are capable of fostering tissue regeneration after transplantation. However, the behavior and physiological role of endogenous MSCs in distal organ repair remain undetermined. In this study, a suite of genetic tools is generated to distinguish MSCs between tissues, and to map their fate in local, proximate, and distal organ repair. By single‐cell RNA‐sequencing, it is found that the transcriptomic profiles of most non‐bone marrow‐derived mesenchymal stromal cells (nonBM‐MSCs) exhibited high similarities, yet differed from that of bone marrow‐derived mesenchymal stromal cells (BM‐MSCs), especially in their less abundant secretome. Fate‐mapping experiments demonstrated that BM‐MSCs do not contribute to the formation of myofibroblasts during fibrosis or cancer‐associated fibroblasts (CAFs) during tumorigenesis. In contrast, MSCs from proximate tissues actively migrated to the site of bone fracture, where they contributed to the formation of fibrocartilaginous soft callus. During injury‐associated inflammation, local MSCs modulated the polarization of tissue‐resident macrophages, whereas BM‐MSCs mobilized monocytes to the site of inflammation. Conditional deletion of *Ccl2* in BM‐MSCs, but not in colon‐resident MSCs, ameliorated colon inflammation and restored body weight after colitis. Thus, injury repair is orchestrated by MSCs from multiple sources, with local, proximate, and distal MSCs acting through different mechanisms.

## Graphical abstract

This study maps mesenchymal stromal cells (MSCs) across multiple tissues, revealing that bone marrow–derived MSCs mobilize monocytes via CCL2 to promote systemic inflammation but do not migrate to distant organs to form fibrotic or tumor stroma. Instead, local MSCs dominate tissue remodeling, suggesting a “two‐hit” model linking systemic inflammation and organ‐specific fibrosis.

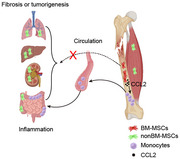

## 1 Introduction

Mesenchymal stromal cells (MSCs) represent a heterogeneous population enriched by depleting hematopoietic and endothelial cells from the stromal‐vascular fraction of dissociated tissues.^[^ ^1^, ^2^ ^]^ The capabilities of self‐renewal and multilineage differentiation led to extensive investigation of their potential to serve as a source of cells for tissue repair and regeneration.^[^ ^3^ ^]^ First isolated from bone marrow (BM), MSCs have since been identified in the perivascular niches of nearly all organs, displaying diverse phenotypes and functional properties.^[^ ^4^, ^5^ ^]^ While the therapeutic potential of in vitro‐expanded MSCs has been widely studied, the in vivo behavior and physiological contributions of endogenous MSCs in distal tissue repair remain poorly understood.^[^ ^6^ ^]^

The ability of MSCs to differentiate into various mesenchymal lineages in vitro has spurred interest in their application for regenerative medicine. Early studies reported that transplanted bone marrow‐derived mesenchymal stromal cells (BM‐MSCs) could give rise to myocytes,^[^ ^7^ ^]^ cardiomyocytes^[^ ^8^ ^]^ and hepatic stellate cells^[^ ^9^ ^]^ at injured sites. Moreover, BM‐MSCs have been implicated as precursors for myofibroblasts during fibrosis and for cancer‐associated fibroblasts (CAFs) within tumors.^[^ ^10^, ^11^ ^]^ Although parabiosis experiments suggest that circulating cells do not directly contribute to tissue fibrosis,^[^ ^12^ ^]^ other studies indicate that parabiosis may not accurately reflect physiological circulation and often underestimates the contribution of circulating cells from donor to recipient.^[^ ^13^, ^14^ ^]^ Notably, recent evidence demonstrates that adipose tissue–derived MSCs can migrate to injured muscle and facilitate regeneration, highlighting the possibility of cross‐tissue MSC mobilization.^[^ ^15^ ^]^ Therefore, these findings underscore persistent uncertainties surrounding the origin, identity, and functional roles of endogenous MSCs in tissue injury and repair.

MSCs exhibit immunomodulatory functions in response to stress, primarily through secreting various factors.^[^ ^16^, ^17^, ^18^, ^19^ ^]^ For instance, BM‐MSC‐derived CSF1 was important for maintaining monocytes within a perivascular niche.^[^ ^20^ ^]^ In response to circulating TLR ligands, BM‐MSCs rapidly expressed CCL2, promoting monocytes trafficking into the bloodstream.^[^ ^21^ ^]^ Colon‐resident MSCs restrained macrophage polarization through TLR4‐p38MAPK‐Cox2 pathway in a dextran sodium sulfate‐induced colitis model.^[^ ^22^ ^]^ In contrast, intravenously transplanted BM‐MSCs migrated to the colon, producing CCL2 and CXCL12 to polarize colon‐resident macrophage during colitis.^[^ ^23^ ^]^ Despite these observations, the immunomodulatory roles of endogenous MSCs, especially those in distal organs, remain unclear.

A major challenge in fate‐mapping MSCs is the widespread expression of most canonical MSC markers across multiple tissues, making it impossible to pinpoint their tissue of origin(s). In this study, we employed single‐cell RNA sequencing (scRNA‐seq) to characterize MSCs across ten adult mouse tissues. Based on the transcriptional signatures of tissue‐specific MSCs, we generated a suite of genetic tools to fate‐map MSCs according to their tissue of origin. *Pdgfra^creER^;Sp7^dre^;R26^ZT1^* mice allowed us to distinguish and simultaneously fate‐map BM‐MSCs and non‐bone marrow‐derived mesenchymal stromal cells (nonBM‐MSCs). *Osx*‐Cre mice mapped the fate of BM‐MSCs, while *Procr*‐CreER mice traced muscle‐resident MSCs but excluded BM‐MSCs. *Tcf21*‐CreER broadly labeled nonBM‐MSCs across tissues while sparing BM‐MSCs. Using this combinatorial toolkit, we systematically examined the behavior and physiological roles of MSCs from local, proximate, and distal origins in diverse tissue injury contexts, including physical damage, fibrosis, inflammation, and tumorigenesis.

## 2 Results

### 2.1 Pdgfra‐CreER Allowed Efficient Fate‐Mapping of MSCs Across Multiple Tissues

**Figure 1:**
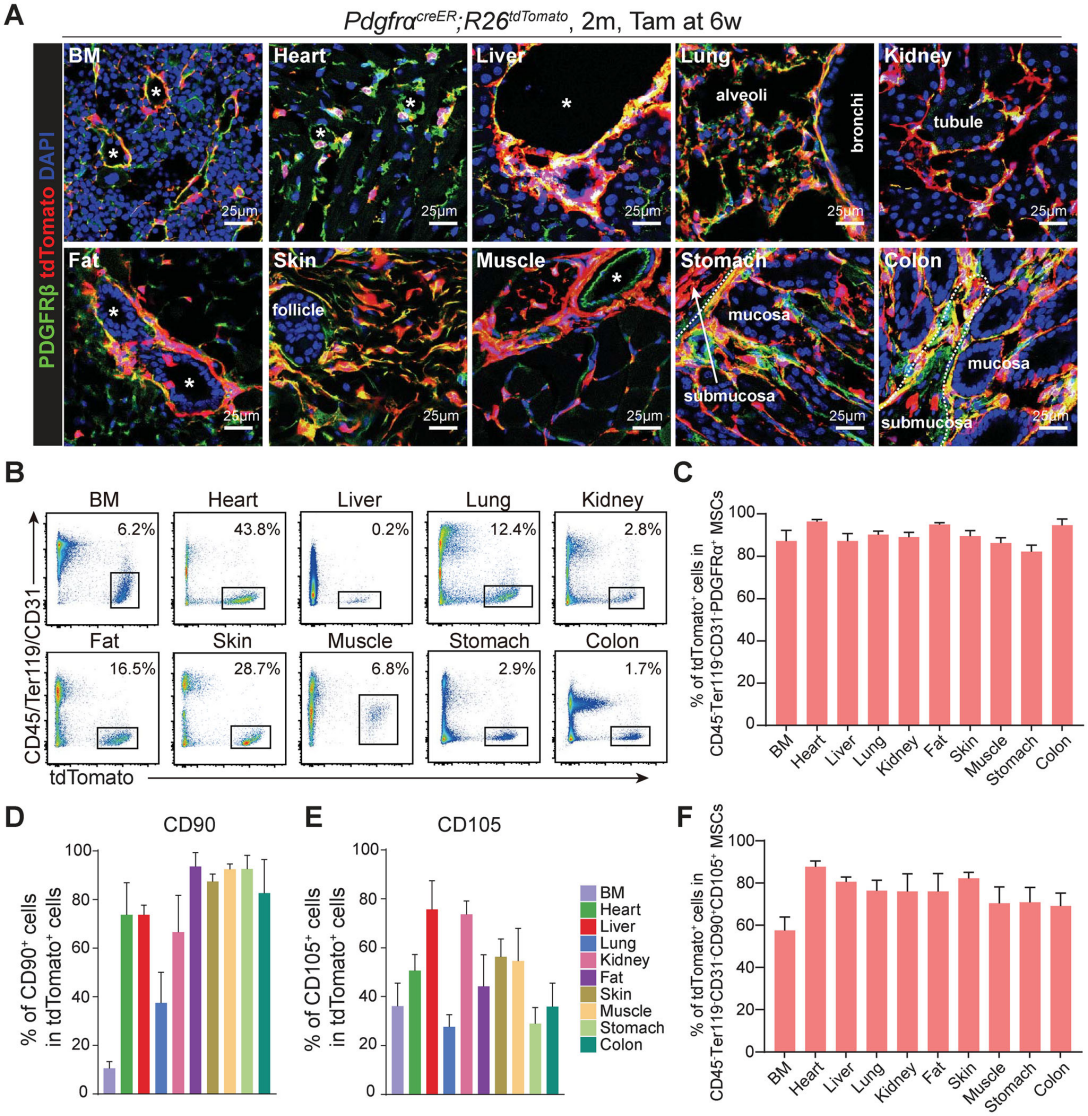
*Pdgfra*‐CreER enabled efficient and specific labeling of MSCs across multiple tissues. A) Confocal imaging revealed efficient labeling of PDGFRβ^+^ stromal cells by tdTomato in different organs of 2‐month‐old *Pdgfra^creER^;R26^tdTomato^* mice treated with tamoxifen at 6 weeks old. Asterisks indicated blood vessels. B) Flow cytometric analysis of enzymatically digested organs showed *Pdgfra*‐CreER selectively recombined in CD45^−^Ter119^−^CD31^−^ non‐hematopoietic, non‐endothelial stromal populations. C) Flow cytometric analysis of enzymatically digested organs showed the percentages of CD45^−^Ter119^−^CD31^−^PDGFRα^+^ MSCs that were tdTomato^+^, in 2‐month‐old *Pdgfra^creER^; R26^tdTomato^* mice treated with tamoxifen at 6‐week‐old (*n* = 3 mice for each group). D,E) Flow cytometric analysis of enzymatically digested organs showed the percentages of CD45^−^Ter119^−^CD31^−^tdTomato^+^ MSCs that expressed CD90 (D) and CD105 (E) (*n* = 4 mice for each group). F) Flow cytometric analysis of enzymatically digested organs showed the percentages of CD45^−^Ter119^−^CD31^−^CD90^+^CD105^+^ MSCs that were tdTomato^+^ (*n* = 4 mice for each group).

To achieve efficient labeling of MSCs in various tissues, we sought a marker that is ubiquitously expressed by MSCs of diverse origins. *Pdgfra* is a well‐established marker for MSCs and their lineages across multiple tissues.^[^ ^24^, ^25^, ^26^ ^]^ We crossed *Pdgfra^creER^* knock‐in mice with *Rosa26^CAG‐loxp‐STOP‐loxp‐tdTomato^* (*R26^tdTomato^*) mice to generate *Pdgfra^creER^;R26^tdTomato^* mice.^[^ ^27^ ^]^ Six‐week‐old *Pdgfra^creER^;R26^tdTomato^* mice were administered with 5 doses of tamoxifen and analyzed 2 weeks later. Confocal imaging confirmed robust labeling of perivascular MSCs in multiple adult tissues, including bone marrow (BM), heart, liver, lung, kidney, subcutaneous fat (fat), skin, muscle, stomach and colon (Figure S1A, Supporting Information). Consistent with their perivascular localization, the majority of tdTomato^+^ cells co‐expressed the mesenchymal marker PDGFRβ in all organs examined (**Figure** **1**A). Flow cytometry further confirmed that *Pdgfra*‐CreER selectively recombined in CD45^−^Ter119^−^CD31^−^ non‐hematopoietic, non‐endothelial stromal populations, labeling over 80% of PDGFRα^+^ MSCs across tissues (Figure 1B,C). While the expression of canonical MSC surface markers CD105 and CD90 (Thy1) varied considerably among MSCs derived from different tissues, the majority of CD45^−^Ter119^−^CD31^−^CD90^+^CD105^+^ MSCs were tdTomato^+^ (Figure 1D–F).^[^ ^1^, ^5^ ^]^ Notably, these two surface markers were not reliable indicators of MSCs in mouse bone marrow, in contrast to their consistent expression in human MSCs, but they proved more dependable in other tissues. These data indicated that *Pdgfra*‐CreER enables efficient and broadly applicable labeling of MSCs across multiple tissues.

### 2.2 BM‐MSCs Displayed a Distinct Transcriptional Profile from nonBM‐MSCs

**Figure 2:**
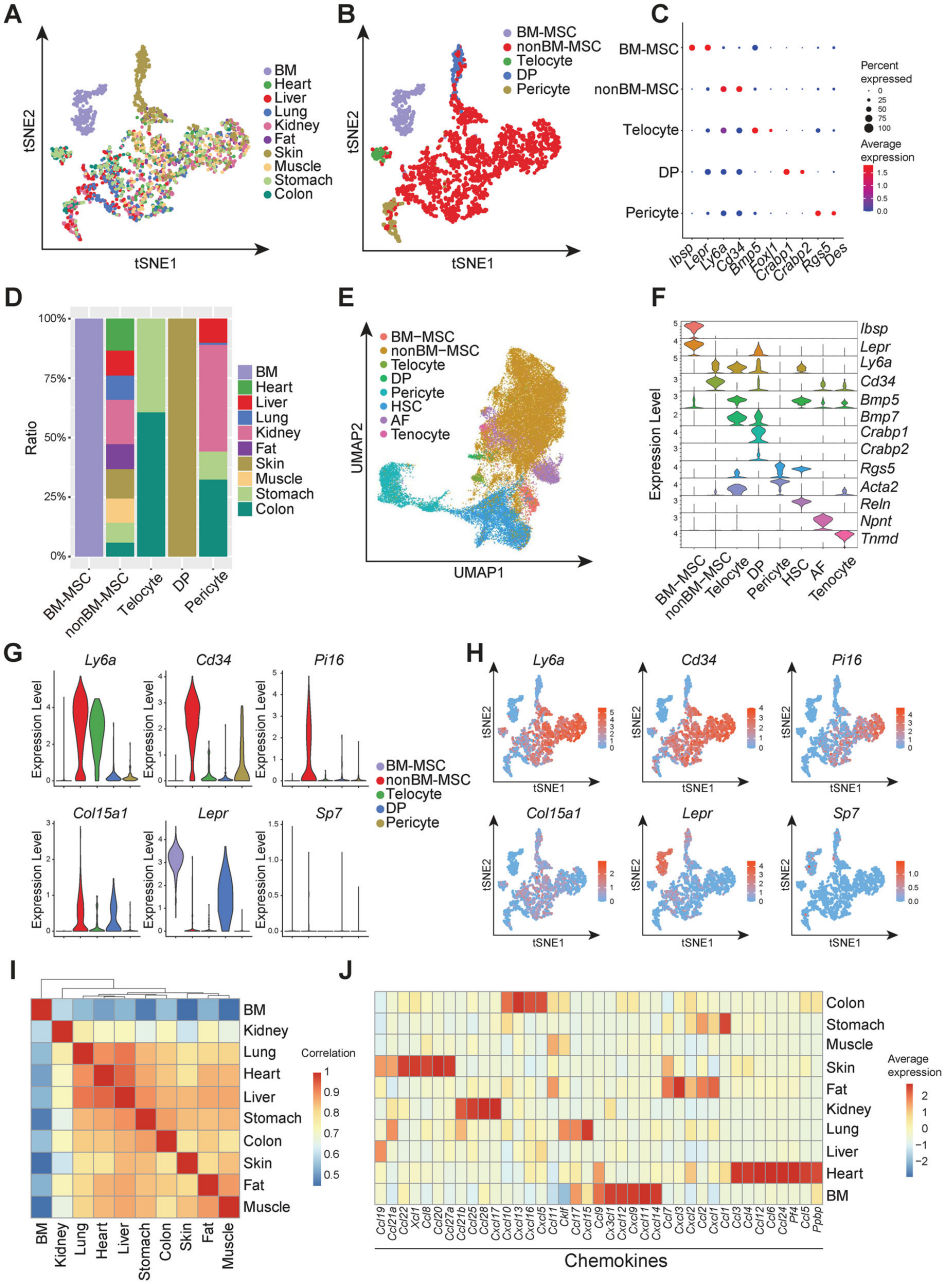
BM‐MSCs displayed a hyper‐rich secretome as compared to nonBM‐MSCs. A,B) t‐SNE visualization of unsupervised clustering of all tdTomato^+^ stromal cells isolated from different organs of 2‐month‐old *Pdgfra^creER^;R26^tdTomato^* mice treated with tamoxifen at 6 weeks old. The colors represented organ origins (A) and different clusters (B). C) Dot plot showing the expression levels of representative marker genes of MSCs across different clusters. D) Bar graph showing the proportions of MSCs from various organs within distinct clusters. E) UMAP visualization of unsupervised clustering of all stromal cells from integrated datasets. The colors represented different clusters. F) Violin plots showing the expression levels of representative marker genes of different clusters from integrated datasets. G,H) Violin plots (G) and feature plots (H) showing the expression levels of indicated genes across different clusters. I) Heatmap showing the Pearson correlation of secreted genes in BM‐MSC and nonBM‐MSC clusters derived from different organs. J) Heatmap showing the average expression levels of chemokines in BM‐MSC and nonBM‐MSC clusters derived from different organs.

To enable tissue‐specific fate‐mapping of MSCs, we first compared the transcriptional signatures of MSCs across diverse tissue origins. We isolated CD45^−^Ter119^−^CD31^−^tdTomato^+^ MSCs from the ten aforementioned tissues by fluorescence‐activated cell sorting (FACS) and subjected them to Smart‐seq2‐based scRNA‐seq. A total of 2412 high‐quality cells passed filtering and were included in downstream analyses. T‐Distributed Stochastic Neighbor Embedding (t‐SNE) revealed that MSCs from most tissues were clustered together (**Figure** **2**A,B). Based on differentially expressed genes and canonical markers, we identified five distinct clusters: *Lepr* ^+^ *Ibsp* ^+^ BM‐MSCs, *Cd34* ^+^ *Ly6a* ^+^ nonBM‐MSCs, *Bmp5* ^+^ *Foxl1* ^+^ telocytes (subepithelial stromal cell in the gastrointestinal tract),^[^ ^28^ ^]^ *Crabp1* ^+^ *Crabp2* ^+^ dermal papilla cells (DP)^[^ ^29^ ^]^ and *Rgs5* ^+^ *Des* ^+^ pericytes (Figure 2C).^[^ ^30^ ^]^ BM‐MSCs formed an independent cluster while MSCs from the other tissues (nonBM‐MSCs) clustered together (Figure 2A,D). To construct a comprehensive atlas of stromal cells, we integrated our dataset with multiple previously published stromal scRNA‐seq datasets.^[^ ^28^, ^31^, ^32^, ^33^, ^34^, ^35^, ^36^, ^37^, ^38^, ^39^ ^]^ This integrated analysis reproduced key features of our dataset, including a distinct BM‐MSC cluster, a universal nonBM‐MSC cluster, a pericyte cluster and several organ‐specific subsets, such as hepatic stellate cells (HSCs) in the liver, alveolar fibroblasts (AFs) in the lung, and tenocytes in muscle (Figure 2E,F; Figure S1B, Supporting Information).^[^ ^40^ ^]^ These data suggested that MSCs from most nonBM tissues share similar transcriptional profiles and BM‐MSCs have a distinct transcriptional profile from nonBM‐MSCs.

Gene ontology (GO) analysis highlighted functional distinctions between BM‐MSCs and nonBM‐MSCs. BM‐MSCs were enriched for pathways related to ossification, bone mineralization, and regulation of chemotaxis, whereas the shared nonBM‐MSC population was enriched for terms associated with cell‐substrate adhesion and cytoskeleton organization (Figure S1C–G, Supporting Information). *Lepr* was predominantly expressed by BM‐MSCs, but also showed low expression in DP^[^ ^41^ ^]^ and a small subset of nonBM‐MSCs (Figure 2G,H). In contrast, stemness‐associated genes such as *Cd34* and *Ly6a*, along with universal fibroblast markers *Pi16* and *Col15a1*, were broadly expressed in nonBM‐MSCs (Figure 2G,H).^[^ ^40^ ^]^ BM‐MSCs lacked *Cd34* expression and rarely expressed *Ly6a*, consistent with previous reports.^[^ ^42^ ^]^

To further investigate whether transcriptomic differences between BM‐MSCs and nonBM‐MSCs are intrinsic or shaped by their local microenvironment, we assessed their plasticity in vitro. We isolated CD45^−^Ter119^−^CD31^−^tdTomato^+^ MSCs from different organs of *Pdgfra^creER^;R26^tdTomato^* mice and cultured them under identical conditions for two weeks. We then analyzed the expression of BM‐MSCs specific markers (*Lepr*, *Cxcl12* and *Kitl*) and nonBM‐MSCs specific markers (*Ly6a* and *Pi16*). RT‐qPCR analysis revealed a partial convergence of their expression profiles. After culturing, BM‐MSCs upregulated the non‐BM marker *Ly6a*, whereas MSCs from the lung, fat, muscle, and colon increased their expression of the BM‐specific markers *Lepr*, *Cxcl12*, and *Kitl* (Figure S1H–L, Supporting Information). Despite these shifts, significant differences between the populations persisted. This suggests that while MSCs are influenced by their surroundings, they also retain stable, intrinsic transcriptional properties from their tissue of origin. Therefore, both cell‐intrinsic factors and the local microenvironment govern MSC identity.

### 2.3 BM‐MSCs had a Hyper‐Rich Secretome as Compared to nonBM‐MSCs

MSCs act as a “drugstore” for injury repair by secreting various factors.^[^ ^43^ ^]^ We compared the secreted gene expression levels of BM‐MSC and nonBM‐MSC clusters from different organs. Pearson correlation analysis revealed that nonBM‐MSCs shared similar secretomic profiles, but were distinct from BM‐MSCs (Figure 2I; Table S1, Supporting Information). BM‐MSCs demonstrated higher expression levels of chemokines, implying a more prominent role in the migration of immune cells (Figure 2J). Consistent with a recent study, stem cell growth factors were highly expressed in BM‐MSCs (Figure S2, Supporting Information).^[^ ^44^ ^]^ BM‐MSCs showed elevated levels of interleukins, including *Il7*, which was crucial for the differentiation and survival of lymphoid progenitors (Figure S2B, Supporting Information). In contrast, inflammatory factors such as *Il6* and *Il33* were primarily expressed by nonBM‐MSCs (Figure S2B, Supporting Information). Meanwhile, MSCs from different organs exhibited diverse expression patterns of growth factors (Figure S2C, Supporting Information). These enhanced trophic and immunomodulatory activities observed in BM‐MSCs indicate that they may offer clinical therapeutic potential.

### 2.4 Genetic Fate‐Mapping Distinguished BM‐MSCs and nonBM‐MSCs

**Figure 3:**
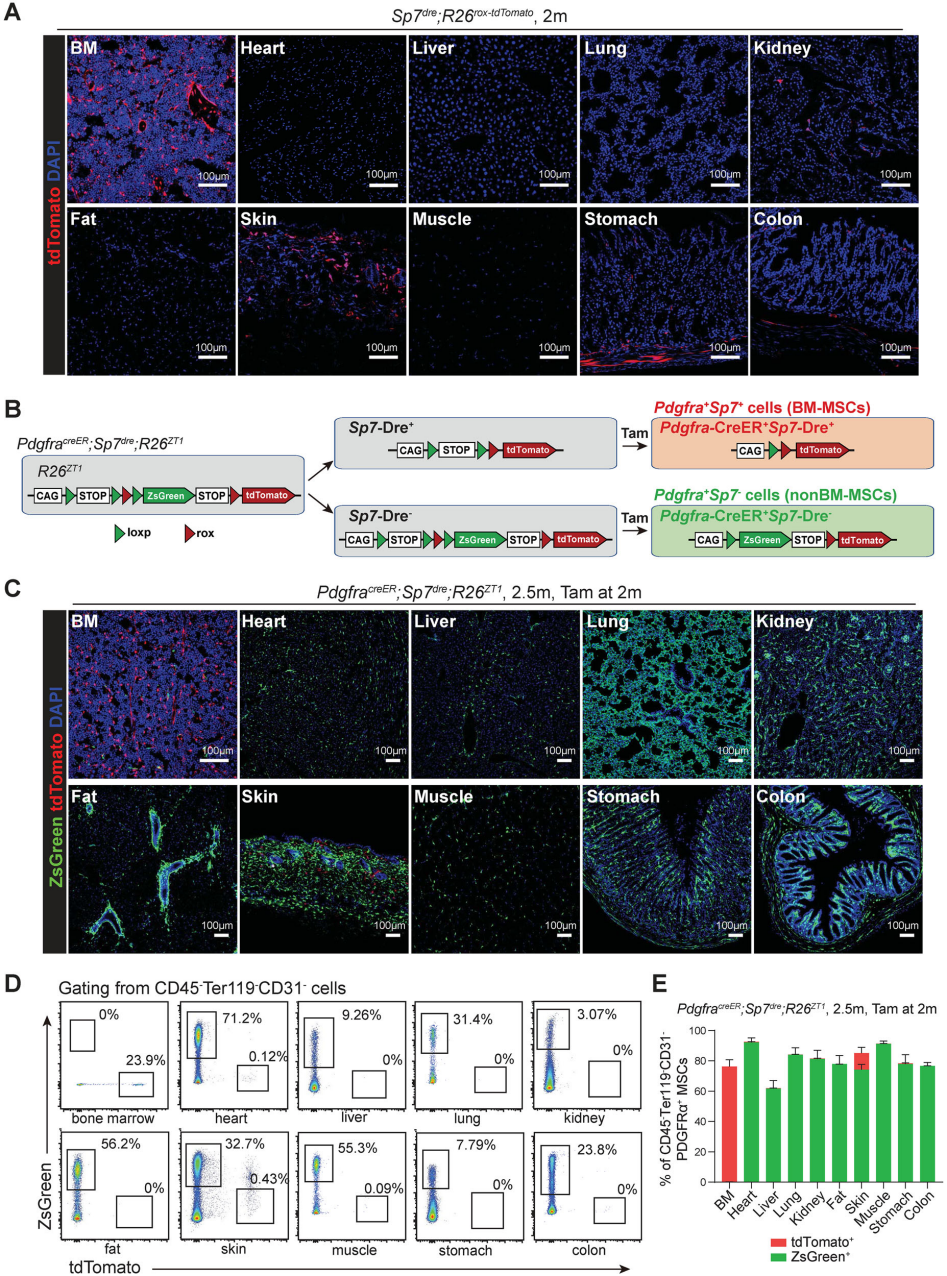
*Pdgfra^creER^;Sp7^dre^;R26^ZT1^* mice distinguished the fates of BM‐MSCs and nonBM‐MSCs. A) Confocal imaging revealed the recombination patterns of *Sp7*‐Dre in different organs. B) Schematic of simultaneous tracing of *Pdgfra^+^Sp7^+^* and *Pdgfra^+^Sp7^−^* cells in *Pdgfra^creER^;Sp7^dre^;R26^ZT1^* mice. C) Confocal imaging of frozen sections from different organs of 2.5‐month‐old *Pdgfra^creER^;Sp7^dre^;R26^ZT1^* mice treated with tamoxifen at 2 months old. D) Flow cytometric analysis of enzymatically digested organs showed the percentages of CD45^−^Ter119^−^CD31^−^ cells that were ZsGreen^+^ and tdTomato^+^, respectively, in 2.5‐month‐old *Pdgfra^creER^;Sp7^dre^;R26^ZT1^* mice treated with tamoxifen at 2 months old. E) Flow cytometric analysis of enzymatically digested organs showed the percentages of CD45^−^Ter119^−^CD31^−^PDGFRα^+^ MSCs that were ZsGreen^+^ and tdTomato^+^, respectively, in 2.5‐month‐old *Pdgfra^creER^;Sp7^dre^;R26^ZT1^* mice treated with tamoxifen at 2 months old (*n* = 5 mice for each group).

Since scRNA‐seq did not identify any genes that could efficiently and strictly distinguish between BM‐MSCs and nonBM‐MSCs, we sought to distinguish them from a developmental perspective. Previous studies indicate that BM‐MSCs arise from embryonic *Osx^+^* osteoprogenitor and *Acan* ^+^ chondrogenic cells.^[^ ^45^, ^46^, ^47^ ^]^ Guided by these findings, we generated *Sp7^dre^* and *Acan^dre^* mouse strains, and found that *Sp7^dre^* labeled BM‐MSCs more efficiently (Figure S3A,B, Supporting Information). Additionally, *Sp7 (Osx)* was absent in MSCs across all adult tissues (Figure 2G,H). Consistent with this, tdTomato expression was nearly undetectable in heart, liver, lung, kidney, fat, muscle, stomach and colon of 2‐month‐old *Sp7^dre^;R26^rox‐tdTomato^* mice (**Figure** **3**A).^[^ ^27^ ^]^ Together, these data confirmed that *Sp7^dre^* specifically labels BM‐MSCs from their embryonic progenitors, providing a robust genetic tool to distinguish them from nonBM‐MSCs.

To achieve orthogonal recombination of *Pdgfra*‐CreER and *Sp7*‐Dre, we crossed them with *Rosa26^loxp‐STOP‐loxp‐rox‐loxp‐ZsGreen‐STOP‐rox‐tdTomato^* (*R26^ZT1^*) mice^[^ ^27^ ^]^ to generate *Pdgfra^creER^;Sp7^dre^;R26^ZT1^* mice (Figure 3B). Upon tamoxifen induction, CreER would recombine in *Pdgfra* ^+^ *Sp7* ^+^ lineages at the *loxp‐STOP‐loxp‐rox‐tdTomato* sequence to generate *Rosa26^loxp‐rox‐tdTomato^*, and in *Pdgfra* ^+^ *Sp7* ^−^ lineages at the *loxp‐STOP‐loxp‐rox‐loxp* sequence to generate *Rosa26^loxp‐ZsGreen‐STOP‐rox‐tdTomato^* (Figure 3B). Therefore, *Pdgfra* ^+^ *Sp7* ^+^ (BM‐MSCs) and *Pdgfra* ^+^ *Sp7* ^−^ lineages (nonBM‐MSCs) are simultaneously marked by tdTomato and ZsGreen, respectively.

Confocal imaging revealed that tdTomato expression (*Pdgfra^+^ Sp7* ^+^ lineages) was robustly detected in BM, but not in the non‐bone marrow (nonBM) organs of *Pdgfra^creER^;Sp7^dre^;R26^ZT1^* mice induced at 2‐month‐old (Figure 3C). In addition, ZsGreen expression (*Pdgfra^+^Sp7* ^−^ lineages) was restricted to MSCs in nonBM organs and only a very small number of ZsGreen⁺ cells were observed in BM (Figure 3C). Consistently, flow cytometry confirmed that tdTomato⁺ MSCs were restricted to the bone marrow, whereas ZsGreen⁺ MSCs were detected exclusively in nonBM organs (Figure 3D). Within the BM, tdTomato was expressed in ≈78% of CD45^−^Ter119^−^CD31^−^PDGFRα^+^ BM‐MSCs and 77% of CD45^−^Ter119^−^CD31^−^LepR^+^ BM‐MSCs (Figure 3E; Figure S3C, Supporting Information). Beyond the central marrow, tdTomato⁺ cells were also enriched in BM‐MSCs located within the trabecular and endosteal regions (Figure S3D, Supporting Information). Two‐month‐old *Pdgfra^creER^;Sp7^dre^;R26^ZT1^* mice did not show any expression of ZsGreen or tdTomato without tamoxifen induction (Figure S3E, Supporting Information). Immunostaining revealed that these tdTomato⁺ cells found in the skin of *Pdgfra^creER^;Sp7^dre^;R26^ZT1^* mice expressed the MSC markers PDGFRβ and DPT, identifying them as a subpopulation of skin‐resident MSCs (Figure S3F,G, Supporting Information). Notably, these cells lacked Sp7 protein expression, suggesting that tdTomato labeling resulted from *Sp7*‐Dre‐mediated recombination during early development, rather than from ongoing *Sp7* expression (Figure S3H, Supporting Information). Parabiosis experiments were performed to demonstrate that these tdTomato⁺ skin‐resident MSCs did not derive from BM‐MSCs via the circulation (Figure S3I,J, Supporting Information). Thus, *Pdgfra^creER^;Sp7^dre^;R26^ZT1^* mice enable simultaneous fate‐mapping of BM‐MSCs and nonBM‐MSCs.

We performed long‐term tracing of the *Pdgfra^creER^;Sp7^dre^;R26^ZT1^* mice treated with tamoxifen at 2 months of age to evaluate the behavior of tdTomato^+^ BM‐MSCs (*Pdgfra^+^Sp7* ^+^ lineages) during aging. Ten months after tamoxifen administration, over 80% Sp7^+^ osteoprogenitors and Perilipin^+^ adipocytes were labeled by tdTomato (Figure S4A–D, Supporting Information). These data suggested that tdTomato^+^ BM‐MSCs (*Pdgfra^+^Sp7* ^+^ lineages) are the main source of new osteoprogenitors and BM adipocytes during bone homeostasis. Confocal imaging and flow cytometric analysis of 12‐month‐old *Pdgfra^creER^;Sp7^dre^;R26^ZT1^* mice showed no tdTomato expression in most nonBM organs, while the frequency and distribution of ZsGreen^+^ nonBM‐MSCs remained largely unchanged (Figure S4E,F, Supporting Information). These data suggested that tdTomato^+^ BM‐MSCs persist long‐term in the BM, but do not migrate to distal organs under homeostasis.

### 2.5 Absence of BM‐MSC‐Derived Myofibroblasts in Distal Organ Fibrosis

**Figure 4:**
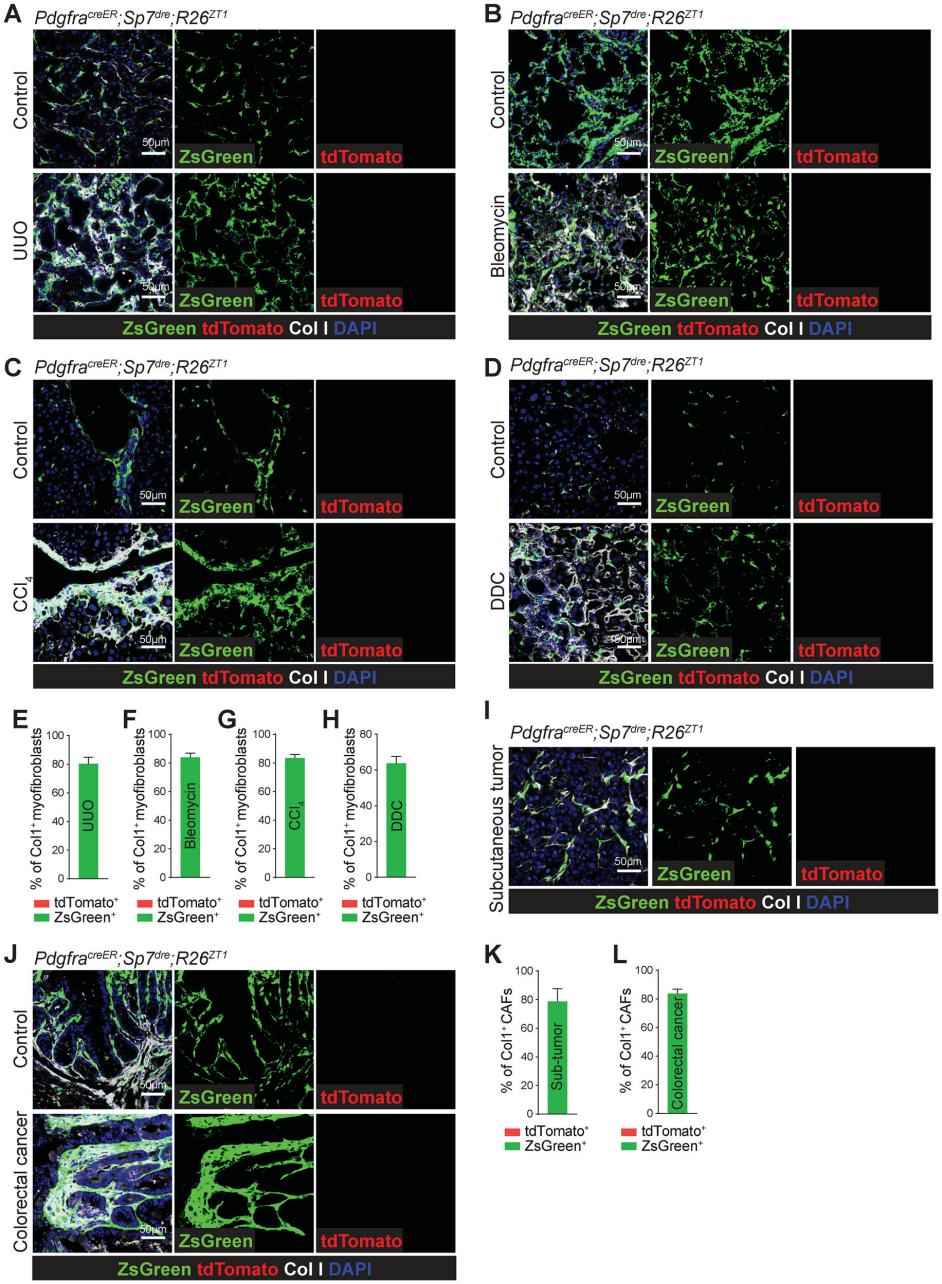
BM‐MSC‐derived myofibroblasts or CAFs were not detected in distal tissue fibrosis or tumors. A) Confocal imaging of kidney sections from normal and UUO‐induced renal fibrotic *Pdgfra^creER^;Sp7^dre^;R26^ZT1^* mice treated with tamoxifen at 2 months old. Myofibroblasts were indicated with anti‐Col1 antibody staining. B) Confocal imaging of lung sections from normal and bleomycin‐induced pulmonary fibrotic *Pdgfra^creER^;Sp7^dre^;R26^ZT1^* mice treated with tamoxifen at 2 months old. Myofibroblasts were indicated with anti‐Col1 antibody staining. C) Confocal imaging of liver sections from normal and CCl_4_‐induced liver fibrotic *Pdgfra^creER^;Sp7^dre^;R26^ZT1^* mice treated with tamoxifen at 2 months old. Myofibroblasts were indicated with anti‐Col1 antibody staining. D) Confocal imaging of liver sections from normal and DDC‐induced liver fibrotic *Pdgfra^creER^;Sp7^dre^;R26^ZT1^* mice treated with tamoxifen at 2 months old. Myofibroblasts were indicated with anti‐Col1 antibody staining. E–H) Quantification of the percentages of DAPI^+^Col1^+^ myofibroblasts that were ZsGreen^+^ and tdTomato^+^ in renal fibrosis (E), pulmonary fibrosis (F), CCl_4_‐induced liver fibrosis (G) and DDC‐induced liver fibrosis (H). *n* = 5 mice from 4 independent experiments. I) Confocal imaging of E0771‐induced subcutaneous tumors from *Pdgfra^creER^;Sp7^dre^;R26^ZT1^* mice treated with tamoxifen at 2 months old. Cancer associated fibroblasts (CAFs) were indicated with anti‐Col1 antibody staining. J) Confocal imaging of normal colons and AOM/DSS‐induced colorectal cancer from *Pdgfra^creER^;Sp7^dre^;R26^ZT1^* mice treated with tamoxifen at 2 months old. CAFs were indicated with anti‐Col1 antibody staining. K,L) Quantification of the percentages of DAPI^+^Col1^+^ CAFs that were ZsGreen^+^ and tdTomato^+^ in subcutaneous tumors (K) and colorectal cancer (L). *n* = 3 mice from 3 independent experiments.

To investigate the contribution of BM‐MSCs to distal organ injury and tissue regeneration, several types of organ fibrosis models were applied to 2.5‐month‐old *Pdgfra^creER^;Sp7^dre^;R26^ZT1^* mice induced at 2 months of age. Seven days after unilateral ureteral obstruction (UUO) induced renal fibrosis (Figure S5A,B, Supporting Information), we found that ≈ 80% of Col1^+^ myofibroblasts (**Figure** **4**A,E), αSMA⁺ myofibroblasts (Figure S6A,I, Supporting Information), and POSTN⁺ myofibroblasts (Figure S6B,J, Supporting Information) in fibrotic kidneys were ZsGreen^+^ (nonBM‐MSCs lineages), but not tdTomato^+^ (BM‐MSCs lineages). Thus, BM‐MSCs (tdTomato^+^) do not migrate to the kidneys to form myofibroblasts in UUO‐induced renal fibrosis, and the majority of myofibroblasts in fibrotic kidneys derive from nonBM‐MSCs (ZsGreen^+^).

Similarly, lung fibrosis was induced using bleomycin (Figure S5C,D, Supporting Information), hepatotoxic liver fibrosis using carbon tetrachloride (CCl_4_) (Figure S5E,F, Supporting Information), and cholestatic liver fibrosis using diethoxycarbonyl‐1,4‐dihydrocollidine (DDC) (Figure S5G,H, Supporting Information). The results showed that 60%‐90% of Col1^+^ myofibroblasts (Figure 4B–H), αSMA⁺ myofibroblasts (Figure S6C–P, Supporting Information), and POSTN⁺ myofibroblasts (Figure S6C–P, Supporting Information) were ZsGreen^+^ in these fibrotic tissues, and none were tdTomato^+^. These data indicated that BM‐MSCs (tdTomato^+^) do not make a detectable contribution to the myofibroblast population during distal organ fibrosis.

### 2.6 Absence of BM‐MSC‐Derived CAFs in Distal Tumors

To investigate the potential contribution of BM‐MSCs to the cancer‐associated fibroblast (CAFs) pool, E0771 breast cancer cells on C57BL/6 background^[^ ^48^ ^]^ were implanted subcutaneously into the groin of 2.5‐month‐old *Pdgfra^creER^;Sp7^dre^;R26^ZT1^* mice induced at 2 months of age. Two weeks later, mice developed visible subcutaneous tumors. Immunostaining revealed the presence of Col1^+^ CAFs throughout the tumors (Figure 4I). About 79% of Col1^+^ CAFs (Figure 4I,K), 88% of αSMA⁺ CAFs (Figure S7B,E, Supporting Information), 82% of POSTN⁺ CAFs (Figure S7C,F, Supporting Information), and 67% of Vimentin⁺ CAFs (Figure S7D,G, Supporting Information) were ZsGreen^+^ whereas no tdTomato^+^ cells were detected in the tumors. These results argue against a significant contribution of circulating BM‐MSCs to the CAF pool in subcutaneous tumors, pointing instead to local or other nonBM‐MSC populations as the principal source.

We also employed an azoxymethane (AOM) and dextran sulfate sodium (DSS) induced colorectal cancer (CRC) model.^[^ ^49^ ^]^ After 90 days of AOM‐DSS treatment, classical CRC occurred in *Pdgfra^creER^;Sp7^dre^;R26^ZT1^* mice (Figure S7A, Supporting Information). Confocal imaging revealed that ≈ 81% of Col1^+^ CAFs (Figure 4J,L), 82% of αSMA⁺ CAFs (Figure S7H,K, Supporting Information), 85% of POSTN⁺ CAFs (Figure S7I,L, Supporting Information), and 87% of Vimentin⁺ CAFs (Figure S7J,M, Supporting Information) were ZsGreen^+^ while no tdTomato^+^ CAFs were detected in the tumors. Our findings indicate that BM‐MSCs do not significantly contribute to the CAF population in colorectal cancer.

### 2.7 Muscle‐Resident MSCs Contributed to Callus Formation after Bone Fracture

**Figure 5:**
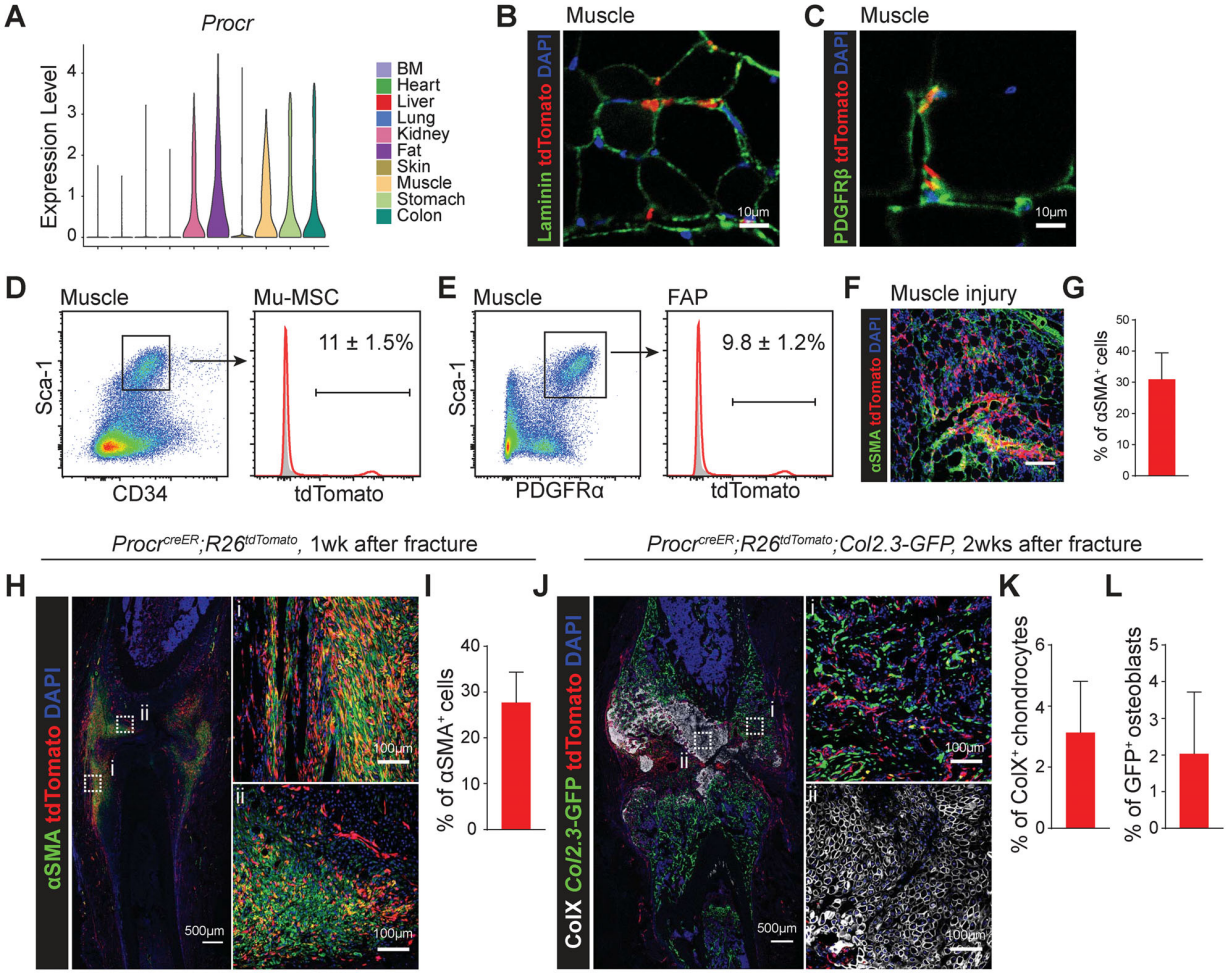
Bone‐proximate muscle MSCs participated in callus formation after bone fracture. A) Violin plot showing the expression levels of *Procr* in stromal cells derived from different organs. B,C) Confocal imaging of muscle sections from 2.5‐month‐old *Procr^creER^;R26^tdTomato^* mice treated with tamoxifen at 2 months old. Anti‐Laminin (B) and Anti‐PDGFRβ (C) staining revealed *Procr*‐CreER labeled tdTomato^+^ cells located in the skeletal muscle interstitium and expressed PDGFRβ. D) Flow cytometric analysis of enzymatically digested muscle cells showed the percentages of CD45^−^Ter119^−^CD31^−^Sca‐1^+^CD34^+^ skeletal muscle MSCs. *n* = 5 mice from 5 independent experiments. E) Flow cytometric analysis of enzymatically digested muscle cells showed the percentages of CD45^−^Ter119^−^CD31^−^Sca‐1^+^PDGFRα^+^ fibro/adipogenic progenitors (FAPs) that were tdTomato^+^. *n* = 4 mice from 4 independent experiments. F,G) Confocal imaging of muscle sections from *Procr^creER^;R26^tdTomato^* mice at 4 days after BaCl_2_ induced muscle injury (F). Activated myofibroblasts were marked by anti‐αSMA antibody staining. The percentages of αSMA^+^ myofibroblasts that were tdTomato^+^ were quantified (G). *n* = 3 mice from 3 independent experiments. H,I) Confocal imaging of femur sections from *Procr^creER^;R26^tdTomato^* mice at 1 week after femur fracture (H). αSMA^+^ fibroblast‐like cells were marked by anti‐αSMA antibody staining. The percentages of αSMA^+^ cells that were tdTomato^+^ were quantified (I). *n* = 5 mice from 5 independent experiments. J–L) Confocal imaging of femur sections from *Procr^creER^;R26^tdTomato^;Col2.3‐GFP* mice at 2 weeks after femur fracture (J). Hypertrophic chondrocytes were marked by anti‐ColX antibody staining. The percentages of ColX^+^ hypertrophic chondrocytes (K) and GFP^+^ osteoblasts (L) that were tdTomato^+^ were quantified. *n* = 3 mice from 3 independent experiments.

We investigated whether MSCs from any nonBM tissues participate in bone repair. Because *Pdgfra^creER^;Sp7^dre^;R26^ZT1^* mice could not distinguish between MSCs from different non‐BM organs, we aimed to develop additional genetic tools for this purpose. From the scRNA‐seq data, we found that protein C receptor (*Procr*) was expressed in MSCs from muscle, fat, kidney, stomach and colon, but absent from other tissues, including BM (**Figure** **5**A).

We constructed *Procr^creER^* knock‐in mice and crossed them with *R26^tdTomato^* mice (Figure S8A, Supporting Information). Two weeks after tamoxifen treatment at 2 months of age, tdTomato expression was detected in the muscle interstitium of *Procr^creER^;R26^tdTomato^* mice, many of which colocalized with PDGFRβ (Figure 5B,C). Flow cytometric analysis revealed ≈ 11% of CD45^−^Ter119^−^CD31^−^Sca‐1^+^CD34^+^ skeletal muscle MSCs^[^ ^50^ ^]^ and 10% of CD45^−^Ter119^−^CD31^−^Sca‐1^+^PDGFRα^+^ fibro/adipogenic progenitors (FAPs)^[^ ^51^ ^]^ were tdTomato^+^ (Figure 5D,E). In addition, some endothelial cells were also tdTomato^+^, accounting for ≈ 20% of VE‐cadherin^+^ endothelial cells (Figure S8B–E, Supporting Information). Furthermore, tdTomato^+^ cells in BM and periosteum were CD31^+^ endothelial cells, instead of MSCs (Figure S8F–I, Supporting Information). After BaCl_2_ induced muscle injury, ≈ 30% of all αSMA^+^ myofibroblasts were labeled by tdTomato, indicating *Procr*‐CreER^+^ muscle MSCs participated in muscle fibrosis after injury (Figure 5F,G). *Procr*‐CreER traced a few CD31^+^ endothelial cells in most other nonBM organs except for the colon, where it traced a subset of perivascular MSCs (Figure S8J, Supporting Information). In conclusion, *Procr*‐CreER distinguishes muscle‐resident MSCs from BM‐MSCs.

We performed femur fracture on 2.5‐month‐old *Procr^creER^;R26^tdTomato^* mice that had been tamoxifen treated at 2 months of age. One week after fracture, soft callus was apparent at the fractured site (Figure 5H). A significant portion of the soft callus was composed of αSMA^+^ fibroblast‐like cells. Approximately 27% of them were labeled by tdTomato (Figure 5I). In contrast, only ≈ 3.1% of ColX^+^ hypertrophic chondrocytes and 2% *Col2.3*‐GFP^+^ osteoblasts were tdTomato^+^ in the callus (Figure 5J–L). Besides, some CD31^+^ endothelial cells at the edge of the callus also expressed tdTomato (Figure S8K, Supporting Information). To rule out the contribution of *Procr*‐CreER traced endothelial cells, we used *Cdh5^creER^;R26^tdTomato^* mice to label *Procr* ^+^ endothelial cells. Flow cytometric analyses showed that ≈ 95% of EPCR⁺(encoded by *Procr*)VE‐cadherin⁺ endothelial cells were tdTomato^+^ (Figure S8L, Supporting Information). Confocal imaging revealed that *Cdh5*‐CreER traced endothelial cells did not contribute to callus formation after bone fracture (Figure S8M, Supporting Information).

To evaluate the potential contribution of *Procr*‐CreER traced MSCs from distal organs to bone regeneration, we performed parabiosis between *Procr^creER^;R26^tdTomato^* mice (CD45.2) and wild type mice (CD45.1). Peripheral blood chimerism was continuously monitored by flow cytometry, and a stabilized femoral fracture were induced in wild‐type parabionts after stable equilibrium was achieved (Figure S8N, Supporting Information). Two weeks post‐fracture, no tdTomato⁺ cells were detected in the fracture callus of wild‐type parabionts (Figure S8O, Supporting Information). These findings indicated that tdTomato^+^ cells from distal organs are not recruited to the injured bone through circulation. Taken together, our results demonstrated that muscle‐derived MSCs, but not MSCs derived from distal organs, contribute to fibrocartilaginous soft callus formation after fracture.

### 2.8 BM‐MSCs‐Derived Chemokine Mobilized Monocytes to the Site of Inflammation

**Figure 6:**
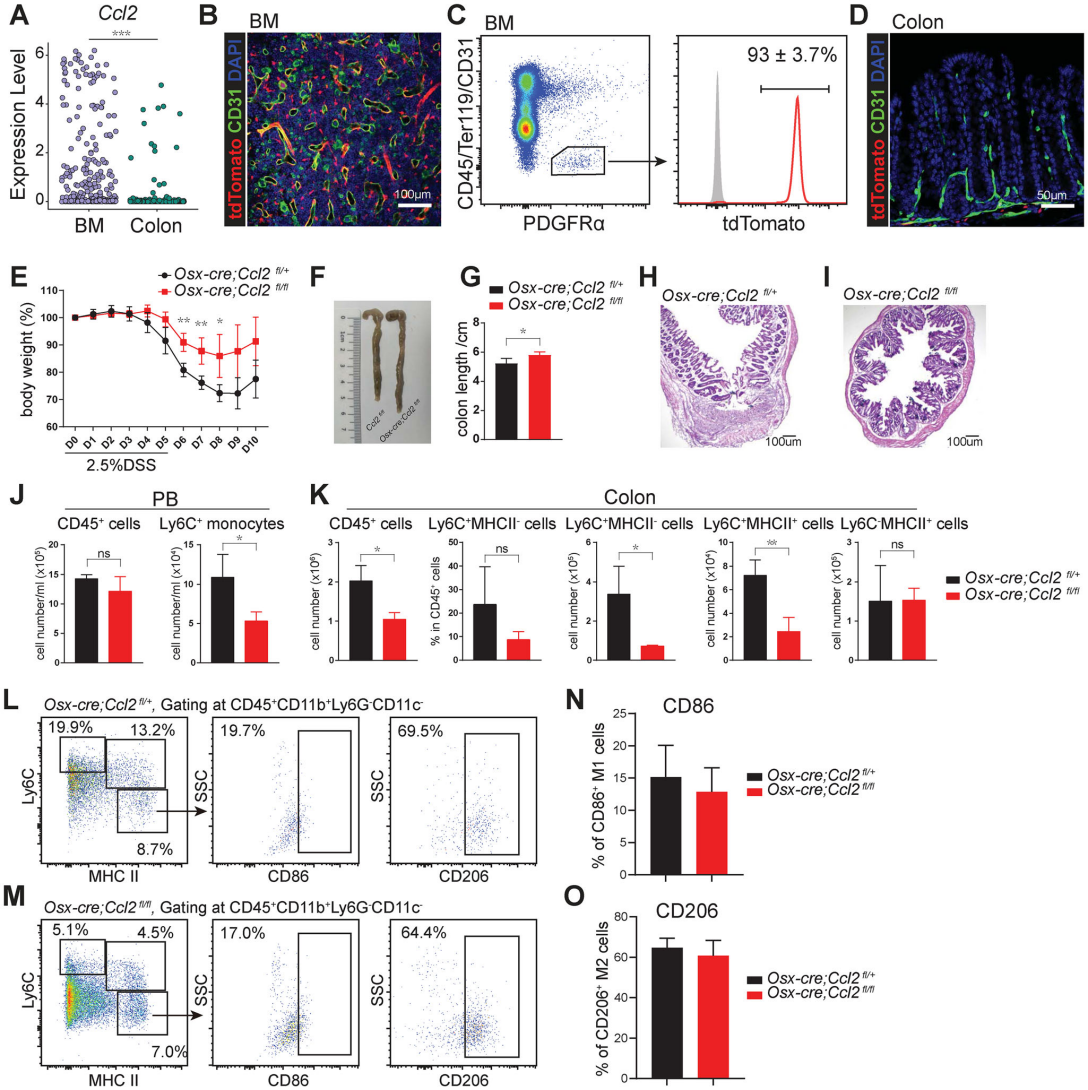
BM‐MSCs‐derived CCL2 promoted colitis at a distance. A) Dot plot showing the expression levels of *Ccl2* in BM and colon stromal cells. B) Confocal imaging of femur sections from 2‐month‐old *Osx‐cre;R26^tdTomato^* mice showed extensive perivascular stromal cell were tdTomato^+^. C) Flow cytometric analysis of enzymatically digested BM cells showed the most CD45^−^Ter119^−^CD31^−^PDGFRα^+^ BM‐MSCs were tdTomato^+^ in 2‐month‐old *Osx‐cre;R26^tdTomato^* mice. D) Confocal imaging of colon sections from 2‐month‐old *Osx‐cre;R26^tdTomato^* mice showed few tdTomato^+^ cells existed in colon. E) Development of colitis was monitored by measuring body weight changes relative to the initial body weight (day 0) in *Osx‐cre;Ccl2^fl/fl^* and control mice following 5 days of 2.5% DSS administration. All data represent mean ± SD from 5–8 mice per time point from 3 independent experiments. Statistical significance was assessed using two‐tailed Student's *t*‐tests. (*p<0.05, **p < 0.01). F,G) Representative images of colons (F) and colon length (G) from *Osx‐cre;Ccl2^fl/fl^* mice and their controls following 5 days of 2.5% DSS administration. H,I) Representative histological images of colons obtained from control mice (H) and *Osx‐cre;Ccl2^fl/fl^* mice (I) following 5 days of 2.5% DSS administration. J,K) Flow cytometric analysis showed the numbers or percentages of CD45^+^ leukocytes, Ly6C^+^ monocytes, Ly6C^+^MHCII^−^ monocytes, Ly6C^+^MHCII^+^ inflammatory monocytes and Ly6C^−^MHCII^+^ macrophages in peripheral blood (J) and colons (K) from *Osx‐cre;Ccl2^fl/fl^* mice and their controls following 5 days of 2.5% DSS administration. All data represent mean ± SD from 3–5 mice from 3 independent experiments. Two‐tailed Student's *t* tests were used to assess the statistical significance of differences between sex‐matched littermates (*p < 0.05, **p < 0.01, ns: not significant). L–O) Flow cytometric analysis (L,M) and quantification (N,O) of the proportions of CD86⁺ (M1‐like) and CD206⁺ (M2‐like) cells within the Ly6C^−^MHCII⁺ macrophage population. All data represent mean ± SD from 6 mice from 4 independent experiments.

MSCs regulate injury‐associated inflammation.^[^ ^52^ ^]^ We introduced a dextran sodium sulfate (DSS)‐induced colitis model to investigate the role of BM‐MSCs in tissue inflammation. According to the expression pattern of *Sp7* (*Osx*) and *Sp7*‐Dre in MSCs (Figure 2G,H; Figure 3A), we used *Osx*‐cre to specifically mark BM‐MSCs (**Figure** **6**B–D; Figure S9A,B, Supporting Information). Our findings demonstrated that tdTomato^+^ BM‐MSCs did not migrate to the colon after DSS treatment (Figure 4J,L).

Monocyte chemotactic protein‐1 (MCP1 or CCL2) is an important chemokine in regulating monocyte mobilization.^[^ ^21^ ^]^ From scRNA‐seq data, we found that *Ccl2* was expressed in BM‐MSCs but was largely absent in colon‐resident MSCs (Figure 6A). We generated *Osx‐cre;Ccl2^fl/fl^* mice to specifically block CCL2 secretion from BM‐MSCs. Under steady state, these mice exhibited normal frequencies and numbers of leukocytes (CD45^+^) and monocytes (CD11b^+^Ly6C^high^) in BM, peripheral blood (PB) and colon (Figure S9C–O, Supporting Information). In DSS‐induced colitis model, DSS treatment disrupted the integrity of the epithelial layer and induced colon inflammation, which resulted in weight loss and shortening of the colon (Figure 6E–G). Meanwhile, DSS exposure accelerated the differentiation of *Hlf⁺* hematopoietic stem and progenitor cells (HSPCs) into monocytes and enhanced their recruitment to the injured colon (Figure S9P, Supporting Information).^[^ ^53^, ^54^ ^]^ Compared with *Osx‐cre;Ccl2^fl/+^* mice, *Osx‐cre;Ccl2^fl/fl^* mice exhibited significantly less weight loss, reduced colon shortening and improved histological outcomes (Figure 6E–I), indicating that *Ccl2* deletion from BM‐MSCs attenuated colitis. Moreover, the number of monocytes in PB decreased in *Osx‐cre;Ccl2^fl/fl^* mice, while the number of leukocytes did not significantly change (Figure 6J). In addition, the colon of *Osx‐cre;Ccl2^fl/fl^* mice exhibited a pronounced reduction in leukocytes, monocytes (Ly6C^+^MHCII^−^), and inflammatory monocytes (Ly6C^+^MHCII^+^), indicating that *Ccl2* deletion in BM‐MSCs attenuates monocyte infiltration (Figure 6K). In contrast, the frequency of monocytes and the number of macrophages (Ly6C^−^MHCII^+^) were unaffected (Figure 6K). We also examined the differentiation of monocytes into pro‐inflammatory (M1‐like) and anti‐inflammatory (M2‐like) macrophages in the colon. Specifically, we analyzed CD86⁺ (M1‐like) and CD206⁺ (M2‐like) cells within the macrophage population (Figure 6L,M). Conditional deletion of *Ccl2* in BM‐MSCs using *Osx*‐cre did not lead to a significant shift in the M1/M2‐like macrophage balance in the inflamed colon (Figure 6N,O). Taken together, these data suggested that BM‐MSCs‐derived CCL2 is a key mediator of monocyte recruitment to sites of inflammation, whereas it exerted minimal impact on the downstream polarization of macrophages.

### 2.9 CCL2 Produced by Colon‐Resident MSCs was Dispensable for the Pathogenesis of Colitis

**Figure 7:**
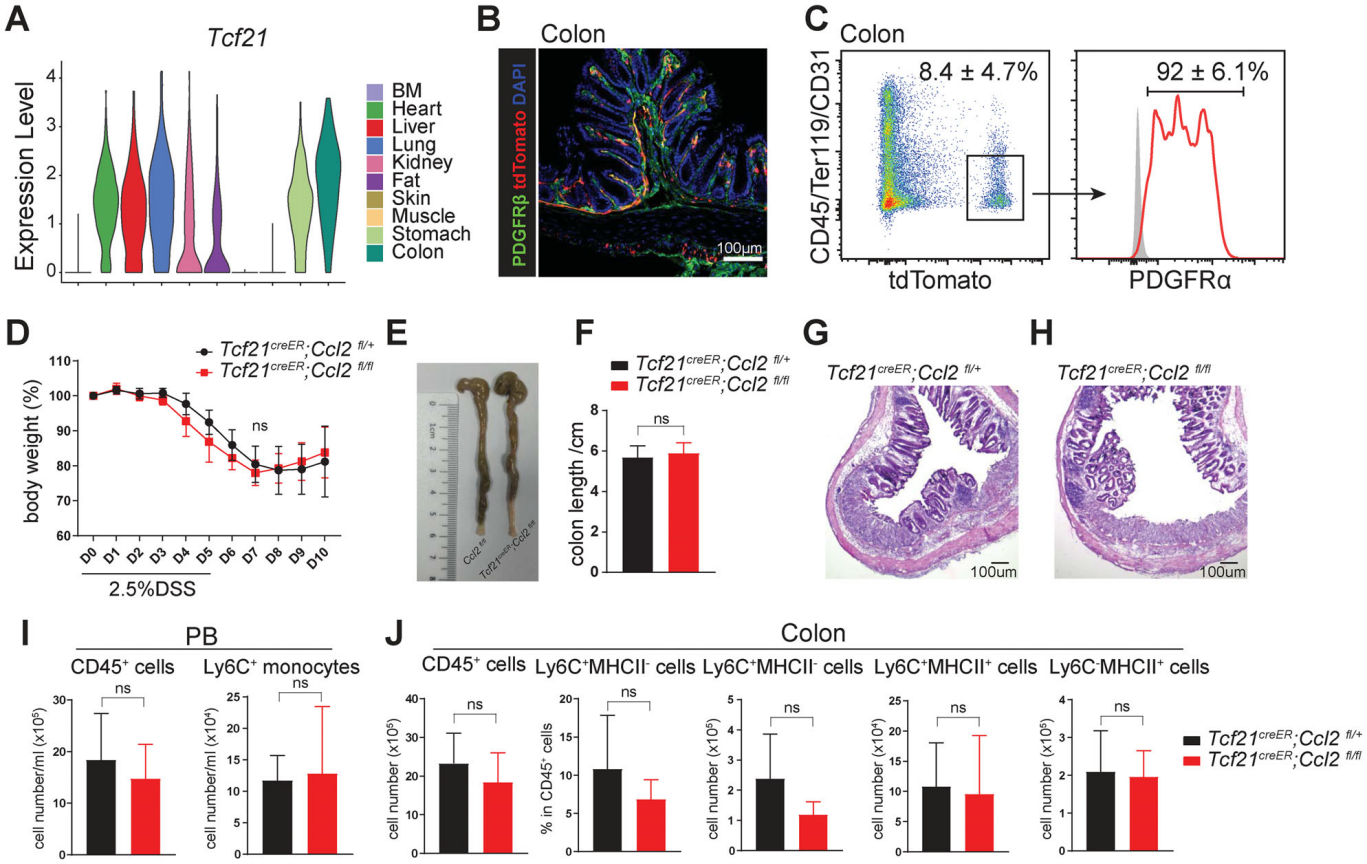
*Ccl2* deletion in colon‐resident MSCs was dispensable for colonic inflammation. A) Violin plot showing the expression levels of *Tcf21* in stromal cells derived from different organs. B) Confocal imaging revealed efficient labeling of PDGFRβ^+^ MSCs by tdTomato in colon of 2.5‐month‐old *Tcf21^creER^;R26^tdTomato^* mice treated with tamoxifen at 2 months old. C) Flow cytometric analysis of enzymatically digested colon non‐epithelial cells show that the majority of tdTomato^+^ expressed PDGFRα in 2.5‐month‐old *Tcf21^creER^;R26^tdTomato^* mice treated with tamoxifen at 2 months old. D) Development of colitis was monitored by measuring body weight changes relative to the initial body weight (day 0) in *Tcf21^creER^;Ccl2^fl/fl^* and control mice with 5 days administration of 2.5% DSS. All data represent mean ± SD from 5–8 mice per time point from 3 independent experiments. The statistical significance of differences at same time points was measured by two‐tailed Student's *t*‐tests (ns: not significant). E,F) Representative images of colons (E) and colon length (F) from *Tcf21^creER^;Ccl2^fl/fl^* mice and their controls following 5 days of 2.5% DSS administration. G,H) Representative histological images of colons obtained from control mice (G) and *Tcf21^creER^;Ccl2^fl/fl^* mice (H) following 5 days of 2.5% DSS treatment. I,J) Flow cytometric analysis showed the numbers or percentages of CD45^+^ leukocytes, Ly6C^+^ monocytes, Ly6C^+^MHCII^−^ monocytes, Ly6C^+^MHCII^+^ inflammatory monocytes and Ly6C^−^MHCII^+^ macrophages in peripheral blood (I) and colons (J) from *Tcf21^creER^;Ccl2^fl/fl^* mice and their controls following 5 days of 2.5% DSS administration. All data represent mean ± SD from 3–5 mice from 3 independent experiments. Two‐tailed Student's *t* tests were used to assess the statistical significance of differences between sex‐matched littermates (ns: not significant).

Colon‐resident MSCs have been reported to regulate colitic inflammation.^[^ ^22^ ^]^ To investigate whether CCL2 secreted by colon‐resident MSCs contributes to the pathogenesis of colitis, we sought to establish a genetic strategy that specifically labels colon‐resident MSCs while sparing BM‐MSCs. Analysis of our scRNA‐seq data revealed that *Tcf21* was robustly expressed in MSCs from the colon and other internal organs, but not in those from the BM (**Figure** **7**A). We generated *Tcf21^creER^;R26^tdTomato^* mice and characterized their reporter expression patterns.^[^ ^55^ ^]^ Confocal imaging demonstrated that tdTomato^+^ cells were selectively present in heart, liver, lung, kidney, stomach and colon (Figure S10A, Supporting Information). In these organs, the majority of tdTomato^+^ cells expressed the mesenchymal marker PDGFRβ (Figure 7B; Figure S10B, Supporting Information). Flow cytometric analysis further showed that *Tcf21*‐CreER marked ≈ 8.4% of non‐epithelial cells in the colon, with the majority of tdTomato⁺ cells co‐expressing PDGFRα (Figure 7C). These findings suggested that *Tcf21^creER^;R26^tdTomato^* mice reliably label colon‐resident MSCs.

To assess the functional role of CCL2 from colon‐resident MSCs, we conditionally deleted *Ccl2* in colon‐resident MSCs using *Tcf21^creER^* mice. Two‐month‐old *Tcf21^creER^;Ccl2^fl/fl^* mice and *Tcf21^creER^;Ccl2^fl/+^* mice were treated with tamoxifen. Quantitative real‐time PCR analyses showed efficient deletion of *Ccl2* in colon‐resident MSCs (Figure S10C, Supporting Information). Two weeks post‐tamoxifen, both groups were subjected to 2.5% DSS in drinking water to induce colitis. No significant differences were observed in weight loss, colon length, or histological severity between the knockout and control groups (Figure 7D–H). In line with these findings, flow cytometric analysis revealed comparable numbers of leukocytes, monocytes, inflammatory monocytes, and macrophages between the two groups (Figure 7I,J). Collectively, these results demonstrated that CCL2 derived from colon‐resident MSCs is dispensable for either immune cell infiltration or the development of DSS‐induced colitis.

## 3 Discussion

MSCs are the focus of intensive efforts worldwide, not only at elucidating their nature and properties but also at developing cell‐based therapies for a diverse range of diseases. Unfortunately, until now the effectiveness of MSC therapy remains controversial. While numerous studies have explored the contributions of exogenously infused MSCs in tissue regeneration,^[^ ^10^, ^11^, ^12^, ^56^ ^]^ the behaviors of endogenous MSCs in the repair of distal tissues remain largely unknown. Furthermore, MSCs exist in virtually all adult organs, where they have been implicated in the repair of their tissue of origin,^[^ ^4^ ^]^ raising additional questions regarding the relationship between tissue‐resident MSCs and bone marrow‐derived MSCs, as well as the division of labor between these populations in tissue repair. Directly addressing these questions has been challenging due to the lack of genetic tools that can specifically track and distinguish MSCs from different tissues. In this study, we developed tissue‐specific genetic tools to investigate the behavior and functional roles of endogenous MSCs, especially BM‐MSCs, in tissue repair and regeneration. Our lineage tracing experiments did not detect a contribution from BM‐MSCs to the formation of myofibroblasts after fibrosis or cancer‐associated fibroblasts after tumor formation, consistent with previous observations from parabiosis experiments.^[^ ^12^, ^57^ ^]^

Our transcriptional profiling of MSCs from ten different organs reveals that BM‐MSCs possess a unique molecular signature, distinguishing them from the largely similar profiles of MSCs from other tissues. This divergence likely reflects their specialized in vivo functions: skeletal homeostasis for BM‐MSCs versus soft tissue maintenance for their non‐BM counterparts. Notably, upon in vitro culture, these distinct expression patterns began to converge, though key differences were retained. Collectively, these findings underscore the plasticity of MSCs and support a model where their identity is shaped by both intrinsic cellular programs and local microenvironmental cues.

Multiple bone‐resident MSC populations, including BM‐MSCs, cambium‐layer and fibrous‐layer periosteal cells, have been shown to be involved in fracture healing.^[^ ^27^, ^58^, ^59^ ^]^ Skeletal muscle cells have been shown to migrate to the site of bone fracture upon transplantation.^[^ ^60^ ^]^ In our current study, we found that *Procr^+^* muscle‐resident MSCs gave rise to a significant portion of αSMA^+^ myofibroblasts in the fibrocartilaginous soft callus during fracture healing, but contributed minimally to the formation of chondrocytes or osteoblasts. Meanwhile, a recent study identified distinct subpopulations of muscle‐resident MSCs with varying adipogenic and mineralizing capacities.^[^ ^61^ ^]^ These results suggested that *Procr^+^* muscle‐resident MSCs represent a functionally specialized progenitor cell population involved in fibrogenesis, but not in mineralization, during fracture repair.

Our observation that proximate muscle‐resident MSCs contribute to bone fracture healing introduces the concept of a ‘regional repair system’ in which adjacent tissues coordinate in response to injury. This has potential implications for understanding complex injuries involving the skin and underlying tissues, such as deep burns, severe trauma, or morphea profunda. The cross‐talk between MSCs from muscle, fat, and dermis may dictate whether the outcome is successful regeneration or pathological fibrosis.^[^ ^62^ ^]^ Modulating this intercellular communication within a specific anatomical region could represent a novel therapeutic approach to prevent excessive scarring and promote functional tissue restoration.

MSCs exhibit versatile immunoregulatory properties, which have been harnessed for the treatment of immune‐mediated disorders.^[^ ^63^, ^64^ ^]^ Bone marrow serves as the primary site for the production and maintenance of most immune cells, whereas tissue‐resident MSCs act as both the initial sensors and key effectors of inflammation. However, due to the lack of genetic tools that distinguish tissue‐specific MSCs, the distinct immunoregulatory roles of BM‐MSCs and tissue‐resident MSCs remain incompletely understood.^[^ ^65^ ^]^ A previous study has shown that colon‐resident MSCs secrete prostaglandin E_2_ (PGE_2_) to suppress M1 macrophage polarization and reduce immune cell infiltration in DSS‐induced colitis.^[^ ^22^ ^]^ Intravenously infused MSCs promote M2 macrophage polarization in the colon.^[^ ^23^ ^]^ Using our newly developed tissue‐specific knockout models, we discovered a distinct role for endogenous BM‐MSCs in colitis. We found they promote inflammation from a distance by mobilizing monocytes to the injured colon. Crucially, the conditional deletion of *Ccl2* in BM‐MSCs did not lead to a detectable shift in the balance of M1‐like and M2‐like macrophages within the inflamed colon. This highlights that tissue‐resident MSCs, endogenous BM‐MSCs, and therapeutically transplanted MSCs each play distinct and non‐overlapping roles in modulating tissue inflammation.

Our discovery of the remote immunomodulatory role of BM‐MSC‐mobilizing monocytes via CCL2 during colitis without directly influencing local macrophage polarization‐provides a potential mechanistic link between systemic inflammation and localized fibrosis. In many autoimmune fibrotic diseases like scleroderma, patients exhibit systemic inflammatory signatures that coincide with organ‐specific fibrosis.^[^ ^66^ ^]^ Our findings suggest a ‘two‐hit’ model where BM‐MSCs contribute to the first hit by fueling systemic monocyte pools, while the second hit is delivered by local tissue‐resident MSCs that respond to these recruited inflammatory cells and initiate a fibrotic cascade. This decoupling of roles suggests that selectively targeting the BM‐MSCs‐CCL2 axis could be a strategy to blunt the systemic inflammatory trigger, potentially preventing or ameliorating fibrotic progression in distal organs like the skin, without interfering with the essential homeostatic functions of local MSCs.

While our study identifies a critical role for CCL2 derived from BM‐MSCs in our colitis model, our data point to a more sophisticated regulatory network for monocyte recruitment. We found that in addition to *Ccl2*, BM‐MSCs also upregulate other potent monocyte chemoattractants, namely *Ccl5*, *Ccl7*, and *Cx3cl1*, while simultaneously downregulating the key bone marrow retention factor *Cxcl12* (Figure S10D, Supporting Information). This concurrent pattern strongly suggests a “push‐pull” mechanism orchestrated by BM‐MSCs: they not only “pull” monocytes into circulation via a suite of attractants but also “push” them out by diminishing niche retention signals. Our findings establish BM‐MSCs as central coordinators of a multi‐component chemokine system that fine‐tunes systemic immune responses during distal inflammation, providing a foundational framework that opens up exciting avenues for future investigation into this complex network.

While this study provides key insights, its conclusions should be considered within the scope of its experimental models. A primary limitation is that our findings are based on acute disease models; the contribution of BM‐MSCs to myofibroblast generation in chronic pathologies warrants further investigation. Additionally, we focused solely on the myofibroblastic CAF (myCAF) subtype, leaving the origins of other CAF populations unresolved. Unraveling the complete lineage map of CAF heterogeneity is therefore a critical next step. We propose that combining our lineage‐tracing system with single‐cell transcriptomics will be essential to definitively chart the developmental pathways of the full spectrum of CAF subtypes.

## 4 Experimental Section

### Mouse Strains

All mice used in this study were maintained on a C57BL/6 background at the Animal Facility of the Center for Excellence in Molecular Cell Science (CEMCS), Chinese Academy of Sciences. The *Pdgfra^creER^*, *R26^tdTomato^*,^[^ ^67^ ^]^ *Sp7^dre^*,^[^ ^27^ ^]^ *R26^rox‐tdTomato^*,^[^ ^68^ ^]^ *R26^ZT1^*, *Tcf21creER*,^[^ ^55^ ^]^ *Cdh5^creER^*,^[^ ^69^ ^]^ *Hlf^creER^*,^[34b]^ *Osx‐cre*,^[^ ^70^ ^]^ and *Ccl2^fl/fl^* ^[^ ^21^ ^]^ mouse strains have been previously described. The *Procr^creER^* knock‐in mouse was generated by inserting the *CreER‐2A‐tdTomato* sequence into the endogenous *Procr* gene locus. These mice were developed by Shanghai Model Organisms Center, Inc. (SMOC). All experimental protocols were approved by the Institutional Animal Care and Use Committees of CEMCS (SIBCB‐S372‐1901‐005).

### Tamoxifen Administration

For induction of CreER activity, tamoxifen (Sigma, T5648) was dissolved in corn oil to a final concentration of 10 mg mL^−1^, and mice were intraperitoneally injected with 100 µL tamoxifen for 5 consecutive days. Additionally, both male and female mice were used for lineage tracing experiments and no sex‐dependent differences were observed.

### Bone Fracture

Mice were anaesthetized by intraperitoneal injection of 150 µL 7% chloral hydrate, and buprenorphine was given 60 min prior to surgery. One leg was shaved and disinfected with 75% ethanol. A 25‐gauge needle was used to puncture the bone marrow through the knee. A stainless‐steel wire (≈0.2 mm in diameter) was inserted into intramedullary canal of the femur to stabilize the impending fracture. Fracture was generated by three‐point bending. The steel wire was removed one week after the fracture, and mice were euthanized for analysis at indicated time point.

### Fibrosis Models

In order to induce renal fibrosis, unilateral ureteral obstruction (UUO) surgery was performed as previously described.^[^ ^12^ ^]^ Briefly, the left kidney was exposed via a flank incision, and the left ureter was ligated with two 4‐0 silk sutures. Mice were euthanized 7 days after obstruction. For the bleomycin‐induced pulmonary fibrosis model, mice were anesthetized with intraperitoneal injection of 150 µL 7% chloral hydrate, and the trachea was exposed through a cervical incision. Bleomycin (Yeasen Biotechnology, 60216ES60) was dissolved in normal saline and administered intratracheally at a dose of 10 mg kg^−1^ body weight using an insulin syringe. Mice were euthanized on day 14, and lungs were perfused with 4% paraformaldehyde (PFA, Solarbio, p1110). Control mice underwent the same procedure but were instilled with normal saline. Toxic liver fibrosis was induced by intraperitoneal injections of carbon tetrachloride (CCl_4_, dissolved in corn oil at a ratio of 1:3) at a dose of 1 µL g^−1^ twice weekly for 6 weeks.^[^ ^56^ ^]^ Control mice received intraperitoneal injections of corn oil (ABCONE, C67366) alone. For cholestatic liver fibrosis, mice were fed a diet containing 0.1% 3,5‐dicarbethoxy‐1,4‐dihydrocollidine (DDC, Sigma, 137030‐25G) for 4 weeks, with control mice fed a standard diet. For BaCl_2_ induced muscle injury, 50 µl of 1.2% BaCl_2_ in sterile 0.9% NaCl was injected into the tibialis anterior (TA) muscles. All experimental procedures were approved by the Institutional Animal Care and Use Committees of CEMCS.

### Tumor Models

The E0771 mouse breast cancer cell line (CRL‐3461, RRID:CVCL_GR23) was obtained from the American Tissue Type Collection (ATCC) in 2018. E0771 cells were cultured in DMEM medium (Gibco, C11995500BT) supplemented with 10% fetal bovine serum (FBS, Gibco, 16000044). Contamination with mycoplasma was ruled out on a quarterly basis using PCR‐based protocols. For the subcutaneous tumor model, cells were resuspended in 100 µl PBS and mixed 1:1 (v/v) with Matrigel (Corning, 354234) immediately before injection. A total of 1 × 10^6^ cells were injected subcutaneously into the groin region of the mice.

The azoxymethane (AOM) and dextran sulfate sodium (DSS)‐induced colorectal cancer (CRC) model were established as previously described.^[^ ^49^ ^]^ Briefly, 6‐ to 8‐week‐old mice were intraperitoneally injected with a single dose of azoxymethane (AOM, 10 mg kg^−1^ body weight) (Sigma, A5486), followed by addition of DSS (MP Biomedicals, 216011080) to the drinking water at a concentration of 2.5% (w/v) for 7 days (first DSS cycle). This was followed by 2 weeks of regular water for recovery, and then DSS cycle was repeated twice. Mice were euthanized on day 90 of the experimental protocol. All experimental procedures were approved by the Institutional Animal Care and Use Committees of CEMCS.

### DSS‐Induced Colitis

Colitis was induced as described previously.^[^ ^23^ ^]^ Briefly, 8‐ to 10‐week‐old mice were treated with 2.5% (w/v) DSS (MP Biomedicals, 216011080) in drinking water for 7 days, followed by normal water for 3 days to allow recovery. Mice were euthanized on day 10 of the experimental protocol.

### Parabiosis

Mice were sedated with controlled isoflurane anesthesia and placed on heating pads to prevent hypothermia. The lateral sides of the mice were then carefully shaved and aseptically prepared. Matched skin incisions were made to the shaved sides and the knee and elbow joints were tied together with nonabsorbable sutures to facilitate coordinated movement. Surgical wound clips and absorbable sutures were then used to join the skins together. Pairs were then kept in clean cages. Flow cytometry analysis of peripheral blood from wildtype parabiont confirmed successful circulatory anastomoses 1 month after parabiosis. A stabilized femoral fracture was then induced to assess the contribution of circulating cells to bone repair.

### Tissue Digestion and Stromal Cell Isolation

Tissue digestion and stromal cells isolation were performed as previously described.^[^ ^71^ ^]^ To isolate bone marrow stromal cells, femur was cut at the proximal end, and a 1‐mL syringe fitted with a 23‐gauge needle containing ice‐cold HBSS (ThermoFisher, 14025134) was inserted into the distal end through the growth plate to gently flush the marrow plug from the cavity. The BM plug was transferred into 1 mL pre‐warmed digestion solution (0.1 mg mL^−1^ DNase I (Sigma, 11284932001), 5 mg mL^−1^ collagenase I (Worthington, LS004197) and 5 mg mL^−1^ collagenase IV (Worthington, LS004189) in HBSS) and incubated in a shaking water bath at 37 °C with agitation at 200 rpm for 45 min. For other tissues, samples were obtained and minced before being placed in a 15‐mL conical tube with 10 mL pre‐warmed digestion solution (0.5 mg mL^−1^ DNase I (Sigma, 11284932001), 1 mg mL^−1^ collagenase I (Worthington, LS004197) and 1 mg mL^−1^ collagenase IV (Worthington, LS004189) in HBSS) and incubated at 37 °C with agitation at 200 rpm for 30 min. After digestion, the single‐cell suspension was filtered through a 40‐µm nylon mesh, and the cells were centrifuged at 1500 rpm for 5 min at 4 °C. The supernatant was discarded, and the cells were washed twice in ice‐cold HBSS (Ca^2+^ and Mg^2+^ free) supplemented with 2% heat‐inactivated fetal bovine serum (ExCell Bio, FSP500). After digestion, the preparations were incubated with Ack for 5 min to remove red blood cells. To enrich for stromal cells, the cell suspension was incubated with the respective fluorophore‐conjugated antibodies (anti‐CD45, anti‐Ter119, anti‐CD31 and anti‐PDGFRα) for 30 min on ice. Fluorescence‐activated cell sorting (FACS) was used to isolate CD45^−^Ter119^−^CD31^−^PDGFRα^+^ stromal cells.

### Flow Cytometry

Freshly prepared cells were incubated with antibodies in 100 µL ice‐cold HBSS (Ca^2+^ and Mg^2+^ free) supplemented with 2% heat‐inactivated fetal bovine serum (FACS buffer). The following antibodies were used to stain cells: anti‐c‐Kit (BioLegend, 105828, 1:200), anti‐Ly6C (BioLegend, 128003, 1:200), anti‐Ly6G (BioLegend, 127623, 1:200), anti‐CD11c (eBioscience, 47‐0114‐80, 1:200), anti‐F4/80 (BD, 566787, 1:200), anti‐MHCII (BioLegend, 107629, 1:200), anti‐CD64 (BioLegend, 139303, 1:200), anti‐CX3CR1 (BioLegend, 149019, 1:200), anti‐CD86 (BioLegend, 105006, 1:200), anti‐CD206 (BioLegend, 141708, 1:200), anti‐CD144 (BD, 562242, 1:200), anti‐CD45 (Biolegend, 103115, 1:200, RRID: AB_312980), anti‐Ter119 (BioLegend, 116223, 1:200, RRID:AB_2137788), anti‐CD31 (BioLegend, 102434, 1:200, RRID:AB_2629683), anti‐CD34 (BioLegend, 128618, 1:100, RRID:AB_2721678), anti‐Sca‐1 (BioLegend, 122512, 1:100, RRID:AB_756197), PDGFRα‐biotin (Biolegend, 135910, 1:100, RRID: AB_2043973), Lepr‐biotin (R&D, BAF497,1:100, RRID: AB_2296953), Streptavidin‐APC (eBioscience, 17‐4317‐82, 1:200) and streptavidin‐brilliant violet 421 (Biolegend, 405225, 1:200). Antibody staining was performed for 30 min on ice. FACS buffer with 1 mg mL^−1^ DAPI was added to samples shortly before analysis to exclude dead cells. Flow cytometric analysis was performed on LSRFortessa (BD Biosciences) or CytoFlex LX (Beckman Coulter). Cell sorting was performed on Aria SORP (BD Biosciences). Data were analyzed using FlowJo software.

### Immunostaining and Confocal Imaging

Freshly dissected tissues were fixed in 4% paraformaldehyde (PFA, Solarbio, p1110) overnight at 4 °C. A shorter incubation period of 3–4 h was used for antigens sensitive to extended fixation. Bones were decalcified in 20% EDTA (Sangon Biotech, 6381‐92‐6) at pH 7.4 for 3–4 days, while soft tissues were cryoprotected with overnight infiltration in 30% sucrose. Then tissues were embedded in optimal cutting temperature (OCT, Thermo scientific, NEG‐50‐6502) compound and stored at −80 °C. OCT‐embedded bones were sectioned using the CryoJane tape‐transfer system (Leica Biosystems, 39475208 and 39475214). As for soft tissues, cryosections of 10 µm in thickness were collected on positively charged slides and stored at −80 °C until use. Sections were blocked in PBS with 5% donkey serum and 0.1% Triton X‐100 for 1 h, followed by overnight incubation at 4 °C with primary antibodies. After washing with PBS three times, sections were incubated with secondary antibodies and DAPI (Sigma, D9542, 1:1000) at 4 °C for 4 h. The antibodies used were as follows: anti‐CD31 (R&D, AF3628, 1:250, RRID: AB_2161028), anti‐PDGFRβ (eBioscience, 14‐1402‐82, 1:100, RRID:AB_467493), anti‐Col1 (ABclonal, A1352, 1:250, RRID:AB_2760381), anti‐DPT (SantaCruz, sc‐376863, 1:300), anti‐periostin (R&D, AF2955, 1:250), anti‐Vimentin (Abcam, ab8069, 1:200), anti‐αSMA (Abcam, ab5694, 1:200, RRID:AB_2223021), anti‐Sp7 (Abcam, ab209484, 1:100, RRID: AB_2892207), donkey anti rabbit Alexa Fluor 488 (Invitrogen, A21206, 1:500, RRID: AB_2535792), donkey anti rabbit Alexa Fluor 647 (Invitrogen, A31573, 1:500, RRID: AB_2536183), donkey anti goat Alexa Fluor 488 (Invitrogen, A11055, 1:500, RRID: AB_2534102) and donkey anti goat Alexa Fluor 647 (Invitrogen, A21447, 1:500, RRID: AB_2535864). Non‐immune immunoglobulins of the same isotype as the primary antibodies served as negative controls. Finally, slides were mounted in ProLong™ Gold anti‐fade reagent (Invitrogen, P36930), and images were acquired on a Leica TCS SP8 WLL or Leica TCS SP8 STED confocal microscope.

### Histological Staining

Paraffin‐embedded tissue sections were subjected to Hematoxylin and Eosin (H&E), Masson's trichrome, and Sirius Red/Fast Green staining following standard protocols. For H&E staining, paraffin‐embedded tissue sections were deparaffinized, rehydrated through graded ethanol, and stained with hematoxylin for 5 min. After rinsing and bluing, sections were counterstained with eosin for 2–3 min, dehydrated, cleared in xylene, and mounted. For Masson's trichrome staining, sections were stained with hematoxylin, Biebrich scarlet‐acid fuchsin, and aniline blue following differentiation with phosphomolybdic‐phosphotungstic acid, allowing visualization of collagen (blue), cytoplasm (red), and nuclei (black). For Sirius Red/Fast Green staining, sections were stained with 0.1% Fast Green FCF (in saturated picric acid) for 10 min, rinsed, and then stained with 0.1% Sirius Red (in saturated picric acid) for 30 min. After washing in 0.5% acetic acid, sections were dehydrated and mounted. This method highlights collagen fibers in red and non‐collagenous proteins in green. Images were acquired on a BX51 microscope (Olympus).

### Single‐Cell Preparation and Sequencing

Single CD45^−^Ter119^−^CD31^−^tdTomato^+^ cells from different organs were isolated and sorted into 96‐well plates (Bio‐Rad, HSP9601) maintained at 4 °C. Cells were then processed using the Smart‐seq2 full‐length single‐cell RNA sequencing protocol.^[^ ^72^, ^73^ ^]^ In brief, cells were lysed in a single‐cell RNA lysis buffer containing 0.2% Triton X‐100, followed by reverse transcription with a template‐switch oligo (TSO) primer and SuperScript II reverse transcriptase (Invitrogen). Whole transcript amplification was performed with KAPA HiFi HotStart ReadyMix (2x; KAPA Biosystems). The resulting PCR products were purified with AMPure XP beads (Agencourt) and quantified with the Qubit dsDNA HS Assay Kit (Thermo Fisher). cDNA libraries were constructed using the Nextera XT DNA Library Preparation Kit (Illumina) and sequenced on an Illumina HiSeq X Ten platform in 150‐bp paired‐end mode by Novogene.

### Single‐Cell RNA Sequencing Data Analysis

The quality of the raw sequencing data was assessed using FASTQC. Reads were mapped to the mouse GRCm38 genome assembly using STAR with default parameters.^[^ ^74^ ^]^ Uniquely aligned reads were counted with RSEM.^[^ ^75^ ^]^ TPM (transcripts per million) gene expression values were used for downstream analysis in Seurat (Version 3.6.4).^[^ ^76^ ^]^ Quality control was performed using the Seurat package. Cells with fewer than 1 000 detected genes or with >10% mitochondrial gene content were excluded. Genes expressed in fewer than 3 cells were also removed from the dataset. To account for batch effects across different samples, Harmony (Version 0.1.1)^[^ ^77^ ^]^ was employed for dataset integration. The top principal components were selected using the elbow method and utilized for dimensionality reduction and unsupervised clustering. t‐Distributed Stochastic Neighbor Embedding (tSNE) and uniform manifold approximation and projection (UMAP) were performed for visualization. Differentially expressed genes (DEGs) across clusters were identified using the FindAllMarkers function in Seurat, applying a one‐sided Wilcoxon rank‐sum test with Bonferroni correction for multiple testing. Genes with an avg_logFC (log fold‐change of the average expression) > 0.5 and an adjusted P < 0.05 were considered significant. DEGs and canonical marker genes were used to identify distinct cell types based on existing literature. The indicated genes for each cluster were used to generate heatmaps. Pathway enrichment analysis was performed using R package clusterProfiler (version 4.0.5).

### Secretory Gene Selection

To identify secretory genes, we retrieved the list of human secreted proteins from the Human Protein Atlas (HPA) secretome dataset (https://www.proteinatlas.org/humanproteome/secretome), which defines the secretome based on protein features including the presence of signal peptides and the absence of transmembrane regions. Human gene symbols were then mapped to their mouse orthologs using the biomaRt package (version 2.50.3) in R. The resulting set of mouse secretory genes was used for downstream expression analysis in BM‐MSCs and non‐BM‐MSCs.

### RNA Extraction and Real‐Time RT‐PCR

Cells were sorted directly into TRIzol (Life Technologies). Following RNA purification, cDNA was synthesized using SuperScript III Reverse Transcriptase (Life Technologies), according to the manufacturers’ instructions. Quantitative real‐time PCR was performed using SYBR green on a LightCycler 96 system (Roche). Relative expression values were calculated for each gene by the comparative CT method with *Gapdh* for normalization. The primers used for qRT‐PCR are listed in Table S2 (Supporting Information).

### Quantification and Statistical Analysis

Data presented in the figures represent results from more than 3 independent experiments conducted on different days using different mice. Results are presented as means ± SD and statistical analyses were generated using GraphPad Prism version 8.2.1. Unpaired two‐tailed Student's *t*‐test was used to compare two groups of data.

## Conflict of Interest

The authors declare no conflict of interest.

## Author Contributions

X.T.T. and B.O.Z. conceived and initiated the project. X.T.T. performed most of the experiments. Y.L.L. and Y.D. participated in some of the confocal imaging experiments and flow cytometry experiments. B.O.Z., S.S.W., and X.T.T. wrote the manuscript and interpreted the data.

## Supporting information



Supporting Information

## Data Availability

The raw data for scRNA‐seq have been deposited at Genome Sequence Archive (https://ngdc.cncb.ac.cn/gsa/) with accession number CRA019287 and are publicly available as of the date of publication. Reviewers can access the raw data at https://ngdc.cncb.ac.cn/gsa/s/lz9A17KI. The processed data for scRNA‐seq have been deposited in the OMIX under OMIX007584 and can be accessed by reviewers at https://ngdc.cncb.ac.cn/omix/preview/FN1rZVJL.
