# Supplementary material for: Mapping the Tissue‐of‐Origins of Mesenchymal Stromal Cells in Injury Repair
Source: Adv Sci (Weinh). 2025 Nov 27;13(3):e09533. doi: 10.1002/advs.202509533 (PMC12806335; doi:10.1002/advs.202509533)
Supplement: Supplementary file 1 — Supporting Information [file ADVS-13-e09533-s001.pdf]

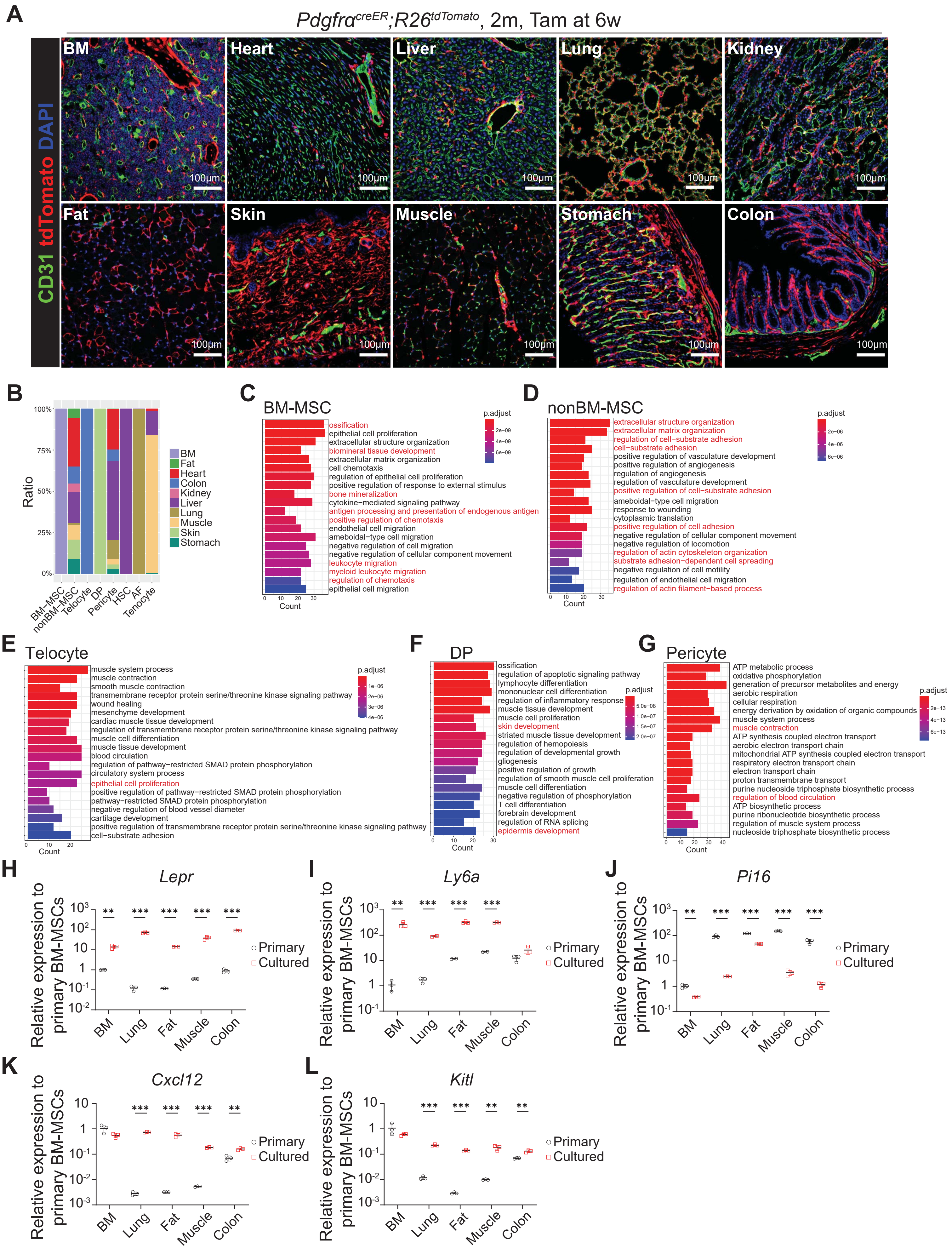
A
Pdgfrα creER;R26 tdTomato, 2m, Tam at 6w
CD31 tdTomato DAPI
BM
Heart
Liver
Lung
Kidney
Fat
Skin
Muscle
Stomach
Colon
100μm
B
Ratio
BM
Fat
Heart
Colon
Kidney
Liver
Lung
Muscle
Skin
Stomach
BM-MSC
nonBM-MSC
Telocyte
DP
Pericyte
HSC
AF
Tenocyte
C
BM-MSC
ossification
epithelial cell proliferation
extracellular structure organization
biomineral tissue development
extracellular matrix organization
cell chemotaxis
regulation of epithelial cell proliferation
positive regulation of response to external stimulus
bone mineralization
cytokine−mediated signaling pathway
antigen processing and presentation of endogenous antigen
positive regulation of chemotaxis
endothelial cell migration
ameboidal−type cell migration
negative regulation of cell migration
negative regulation of cellular component movement
leukocyte migration
myeloid leukocyte migration
regulation of chemotaxis
epithelial cell migration
Count
p.adjust
D
nonBM-MSC
extracellular structure organization
extracellular matrix organization
regulation of cell−substrate adhesion
cell−substrate adhesion
positive regulation of vasculature development
positive regulation of angiogenesis
regulation of angiogenesis
regulation of vasculature development
positive regulation of cell−substrate adhesion
ameboidal−type cell migration
response to wounding
cytoplasmic translation
positive regulation of cell adhesion
negative regulation of cellular component movement
negative regulation of locomotion
regulation of actin cytoskeleton organization
substrate adhesion−dependent cell spreading
negative regulation of cell motility
regulation of endothelial cell migration
regulation of actin filament−based process
E
Telocyte
muscle system process
muscle contraction
smooth muscle contraction
transmembrane receptor protein serine/threonine kinase signaling pathway
wound healing
mesenchyme development
cardiac muscle tissue development
regulation of transmembrane receptor protein serine/threonine kinase signaling pathway
muscle cell differentiation
muscle tissue development
blood circulation
regulation of pathway−restricted SMAD protein phosphorylation
circulatory system process
epithelial cell proliferation
positive regulation of pathway−restricted SMAD protein phosphorylation
pathway−restricted SMAD protein phosphorylation
negative regulation of blood vessel diameter
cartilage development
positive regulation of transmembrane receptor protein serine/threonine kinase signaling pathway
cell−substrate adhesion
F
DP
ossification
regulation of apoptotic signaling pathway
lymphocyte differentiation
mononuclear cell differentiation
regulation of inflammatory response
muscle tissue development
muscle cell proliferation
skin development
striated muscle tissue development
regulation of hemopoiesis
regulation of developmental growth
gliogenesis
positive regulation of growth
regulation of smooth muscle cell proliferation
muscle cell differentiation
negative regulation of phosphorylation
T cell differentiation
forebrain development
regulation of RNA splicing
epidermis development
G
Pericyte
ATP metabolic process
oxidative phosphorylation
generation of precursor metabolites and energy
aerobic respiration
cellular respiration
energy derivation by oxidation of organic compounds
muscle system process
muscle contraction
ATP synthesis coupled electron transport
aerobic electron transport chain
mitochondrial ATP synthesis coupled electron transport
respiratory electron transport chain
electron transport chain
proton transmembrane transport
purine nucleoside triphosphate biosynthetic process
regulation of blood circulation
ATP biosynthetic process
purine ribonucleotide biosynthetic process
regulation of muscle system process
nucleoside triphosphate biosynthetic process
H
Lepr
I
Ly6a
J
Pi16
K
Cxcl12
L
Kitl
Relative expression to primary BM-MSCs
Primary
Cultured
BM
Lung
Fat
Muscle
Colon

Figure S1

Figure S1. ScRNA-seq analysis of MSCs across ten adult tissues.

(A) Confocal imaging revealed efficient labeling of perivascular stromal cells by tdTomato in different organs of 2-month-old *Pdgfra*^{creER};*R26*^{tdTomato} mice treated with tamoxifen at 6 weeks old. Endothelial cells were marked by anti-CD31 antibody staining.

(B) Bar graph showing the proportions of stromal cells from various organs within distinct clusters in integrated datasets.

(C-G) Gene ontology (GO) analysis showed the top 20 enriched pathways of differentially expressed genes (DEGs) in BM-MSC (C), nonBM-MSC (D), telocyte (E), dermal papilla (DP) (F) and pericyte cluster (G).

(H-L) Relative mRNA expression levels of *Lepr* (H), *Ly6a* (I), *Pi16* (J), *Cxcl12* (K) and *Kitl* (L) in primary and *in vitro*–cultured CD45^{-}Ter119^{-}CD31^{-}tdTomato^{+} MSCs sorted from 2.5-month-old *Pdgfra*^{creER};*R26*^{tdTomato} mice treated with tamoxifen at 2 months of age. All data represent mean ± SD from 3 mice from 3 independent experiments. Two-tailed Student's *t* tests were used to assess the statistical significance (**$p < 0.01$, ***$p < 0.001$).

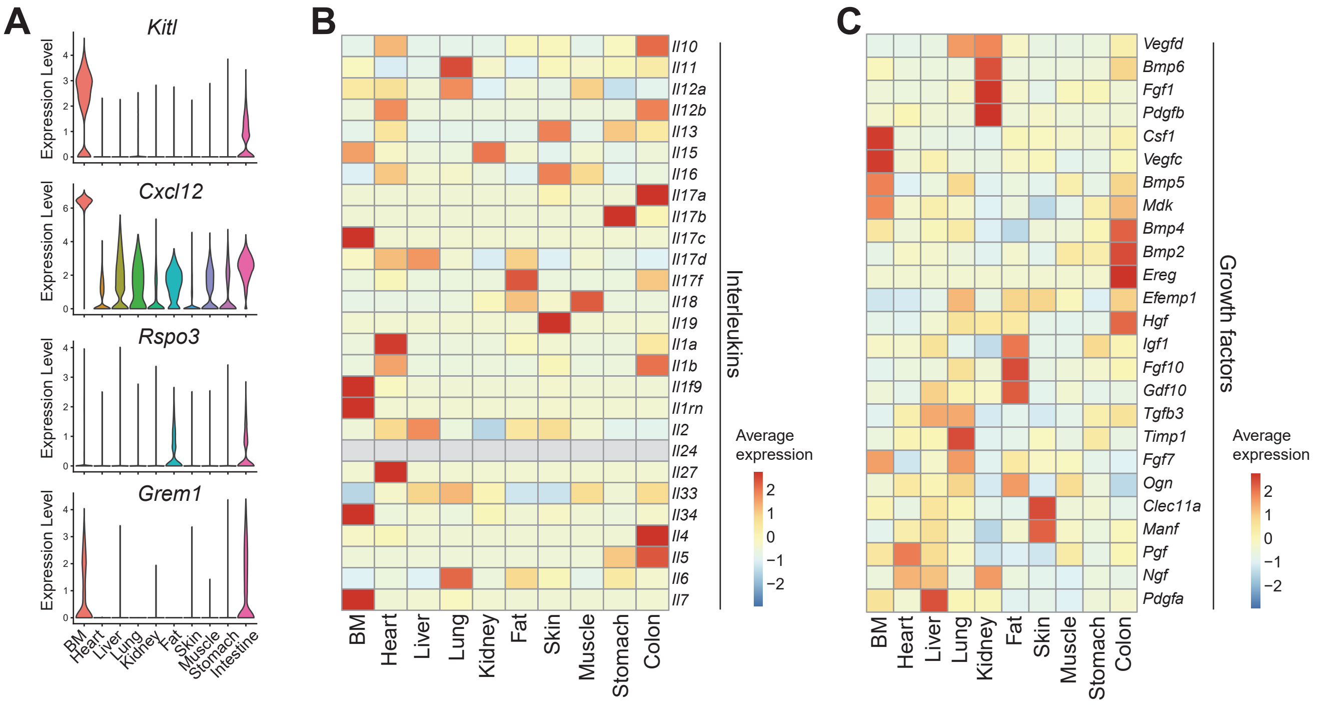

A
Kitl
Cxcl12
Rspo3
Grem1
Expression Level
BM
Heart
Liver
Lung
Kidney
Fat
Skin
Muscle
Stomach
Intestine
B
Il10
Il11
Il12a
Il12b
Il13
Il15
Il16
Il17a
Il17b
Il17c
Il17d
Il17f
Il18
Il19
Il1a
Il1b
Il1f9
Il1rn
Il2
Il24
Il27
Il33
Il34
Il4
Il5
Il6
Il7
Interleukins
Average expression
2
1
0
−1
−2
BM
Heart
Liver
Lung
Kidney
Fat
Skin
Muscle
Stomach
Colon
C
Vegfd
Bmp6
Fgf1
Pdgfb
Csf1
Vegfc
Bmp5
Mdk
Bmp4
Bmp2
Ereg
Efemp1
Hgf
Igf1
Fgf10
Gdf10
Tgfb3
Timp1
Fgf7
Ogn
Clec11a
Manf
Pgf
Ngf
Pdgfa
Growth factors
Average expression
2
1
0
−1
−2
BM
Heart
Liver
Lung
Kidney
Fat
Skin
Muscle
Stomach
Colon

Figure S2

Figure S2. BM-MSCs showed distinct secretory genes from nonBM-MSCs.

(A) Violin plots showing the expression levels of niche factors in stromal cells derived from different organs.

(B,C) Heatmaps showing the expression levels of interleukins (B) and growth factors (C) in stromal cells derived from different organs.

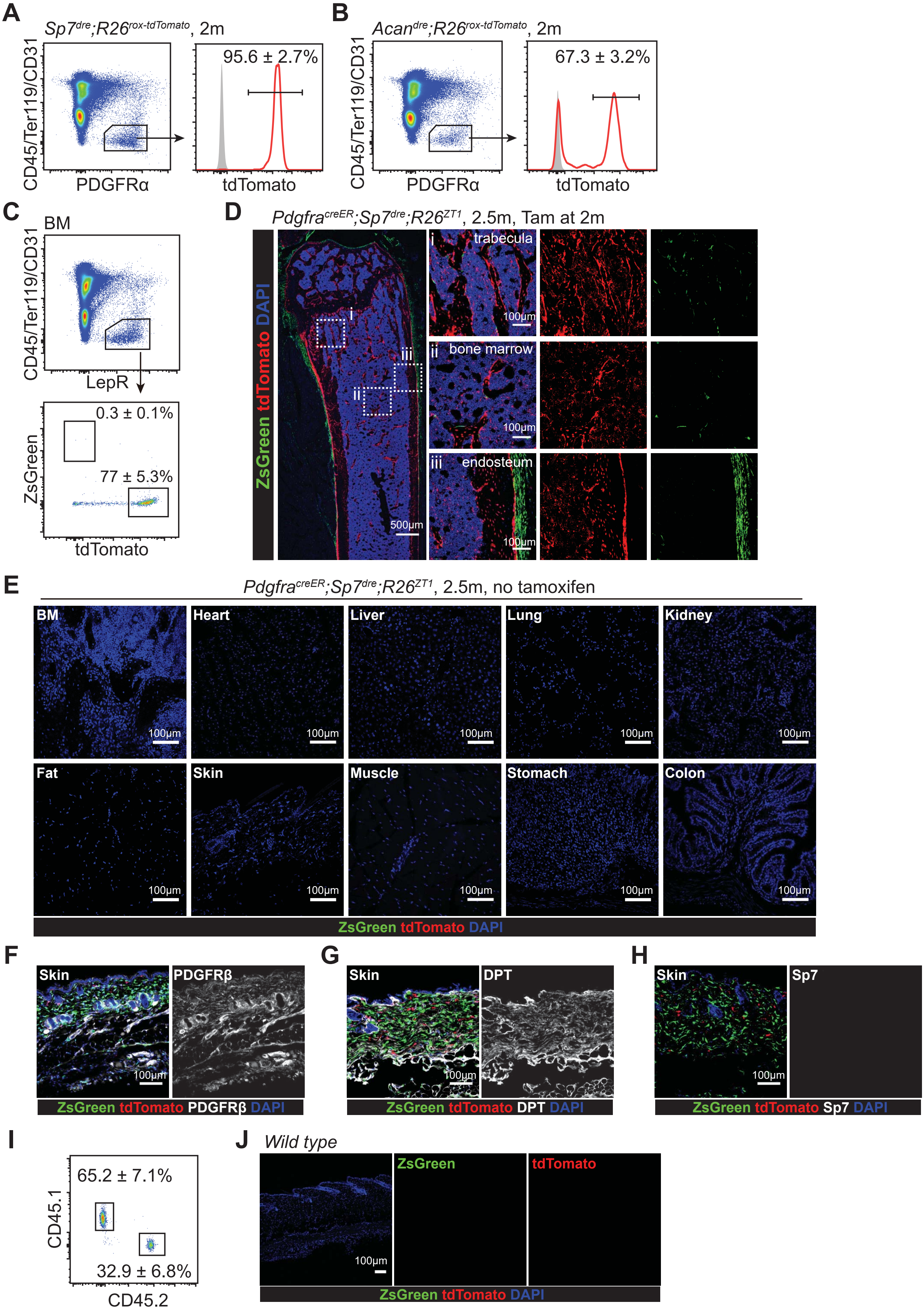
A
Sp7dre;R26rox-tdTomato, 2m
CD45/Ter119/CD31
PDGFRα
95.6 ± 2.7%
tdTomato
B
Acandre;R26rox-tdTomato, 2m
CD45/Ter119/CD31
PDGFRα
67.3 ± 3.2%
tdTomato
C
BM
CD45/Ter119/CD31
LepR
0.3 ± 0.1%
77 ± 5.3%
ZsGreen
tdTomato
D
PdgfracreER;Sp7dre;R26ZT1, 2.5m, Tam at 2m
ZsGreen tdTomato DAPI
i trabecula
ii bone marrow
iii endosteum
500μm
100μm
E
PdgfracreER;Sp7dre;R26ZT1, 2.5m, no tamoxifen
BM
Heart
Liver
Lung
Kidney
Fat
Skin
Muscle
Stomach
Colon
100μm
ZsGreen tdTomato DAPI
F
Skin
PDGFRβ
ZsGreen tdTomato PDGFRβ DAPI
G
Skin
DPT
ZsGreen tdTomato DPT DAPI
H
Skin
Sp7
ZsGreen tdTomato Sp7 DAPI
I
65.2 ± 7.1%
CD45.1
32.9 ± 6.8%
CD45.2
J
Wild type
ZsGreen
tdTomato
ZsGreen tdTomato DAPI

Figure S3

Figure S3. Characterization of the *Pdgfra^{creER};Sp7^{dre};R26^{ZT1}* mice.

(A,B) Flow cytometric analysis of enzymatically dissociated BM from 2-month-old *Sp7^{dre};R26$^{rox\text{-}tdTomato}$* mice (A) and *Acan^{dre};R26$^{rox\text{-}tdTomato}$* mice (B) revealed efficient labeling of CD45$^-$Ter119$^-$CD31$^-$PDGFRα$^+$ BM-MSCs.

(C) Flow cytometric analysis of enzymatically digested bone marrow cells showed the percentages of CD45$^-$Ter119$^-$CD31$^-$LepR$^+$ BM-MSCs that were ZsGreen$^+$ or tdTomato$^+$ in 2.5-month-old *Pdgfra^{creER};Sp7^{dre};R26^{ZT1}* mice treated with tamoxifen at 2 months old.

(D) Confocal imaging of femur sections from 2.5-month-old *Pdgfra^{creER};Sp7^{dre};R26^{ZT1}* mice treated with tamoxifen at 2 months of age, showing the distribution of ZsGreen$^+$ and tdTomato$^+$ BM-MSCs at different regions.

(E) Confocal imaging of *Pdgfra^{creER};Sp7^{dre};R26^{ZT1}* mice without tamoxifen induction showing no detectable ZsGreen or tdTomato expression.

(F,G) Confocal imaging of skin sections from 2.5-month-old *Pdgfra^{creER};Sp7^{dre};R26^{ZT1}* mice at 2 weeks postinduction revealed tdTomato$^+$ cells expressed MSC markers PDGFRβ (F) and DPT (G).

(H) Confocal imaging of skin sections from 2.5-month-old *Pdgfra^{creER};Sp7^{dre};R26^{ZT1}* mice at 2 weeks postinduction revealed tdTomato$^+$ cells did not express Sp7.

(I) Flow cytometry analysis of the peripheral blood from the wild type parabionts. *n* = 3 mice per condition from 3 independent experiments.

(J) Confocal imaging of the skin sections from the wild type parabionts.

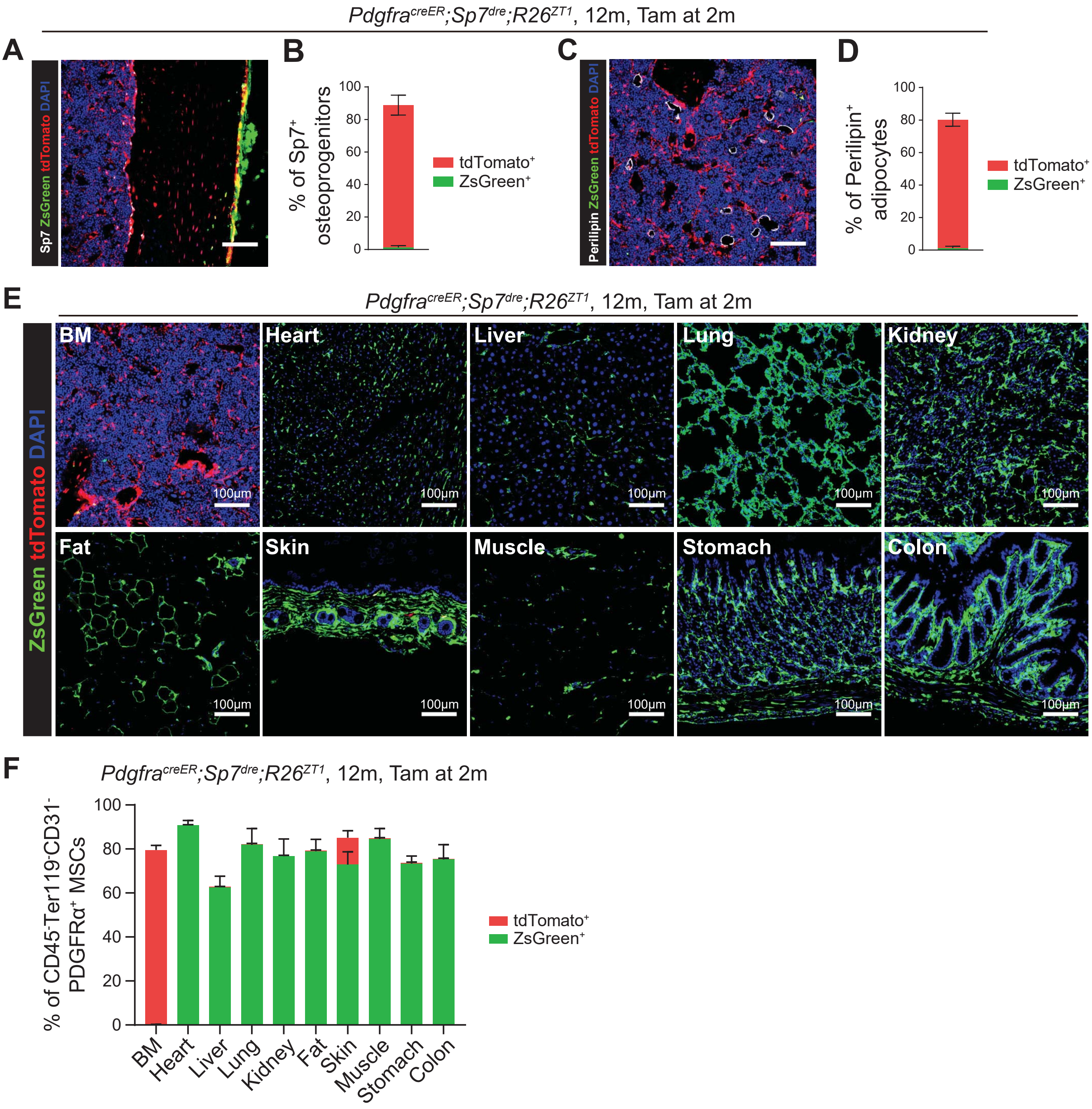

Pdgfra creER;Sp7 dre;R26 ZT1, 12m, Tam at 2m
A
Sp7 ZsGreen tdTomato DAPI
B
% of Sp7+ osteoprogenitors
100
80
60
40
20
0
tdTomato+
ZsGreen+
C
Perilipin ZsGreen tdTomato DAPI
D
% of Perilipin+ adipocytes
100
80
60
40
20
0
tdTomato+
ZsGreen+
E
Pdgfra creER;Sp7 dre;R26 ZT1, 12m, Tam at 2m
ZsGreen tdTomato DAPI
BM
Heart
Liver
Lung
Kidney
Fat
Skin
Muscle
Stomach
Colon
100µm
F
Pdgfra creER;Sp7 dre;R26 ZT1, 12m, Tam at 2m
% of CD45-Ter119-CD31- PDGFRα+ MSCs
100
80
60
40
20
0
BM
Heart
Liver
Lung
Kidney
Fat
Skin
Muscle
Stomach
Colon
tdTomato+
ZsGreen+

Figure S4

Figure S4. BM-MSCs did not migrate to distal organs during aging.

(A-D) Confocal imaging of femur sections from 12-month-old *Pdgfra*^{creER}*;Sp7*^{dre}*;R26*^{ZT1} mice treated with tamoxifen at 2 months of age. Osteoprogenitors were marked by anti-Sp7 antibody staining (A). Bone marrow adipocytes were marked by anti-Perilipin antibody staining (C). The percentages of Sp7^{+} osteoprogenitors (B) and Perilipin^{+} adipocytes (D) that were tdTomato^{+} or ZsGreen^{+} were quantified. *n* = 3 mice from 3 independent experiments.

(E) Confocal imaging of frozen sections from different organs of 12-month-old *Pdgfra*^{creER}*;Sp7*^{dre}*;R26*^{ZT1} mice treated with tamoxifen at 2 months of age.

(F) Flow cytometric analysis of enzymatically digested organs showed the percentages of CD45^{-}Ter119^{-}CD31^{-}PDGFRα^{+} MSCs that were ZsGreen^{+} and tdTomato^{+}, respectively, in 12-month-old *Pdgfra*^{creER}*;Sp7*^{dre}*;R26*^{ZT1} mice treated with tamoxifen at 2 months old. *n* = 4 mice from 3 independent experiments.

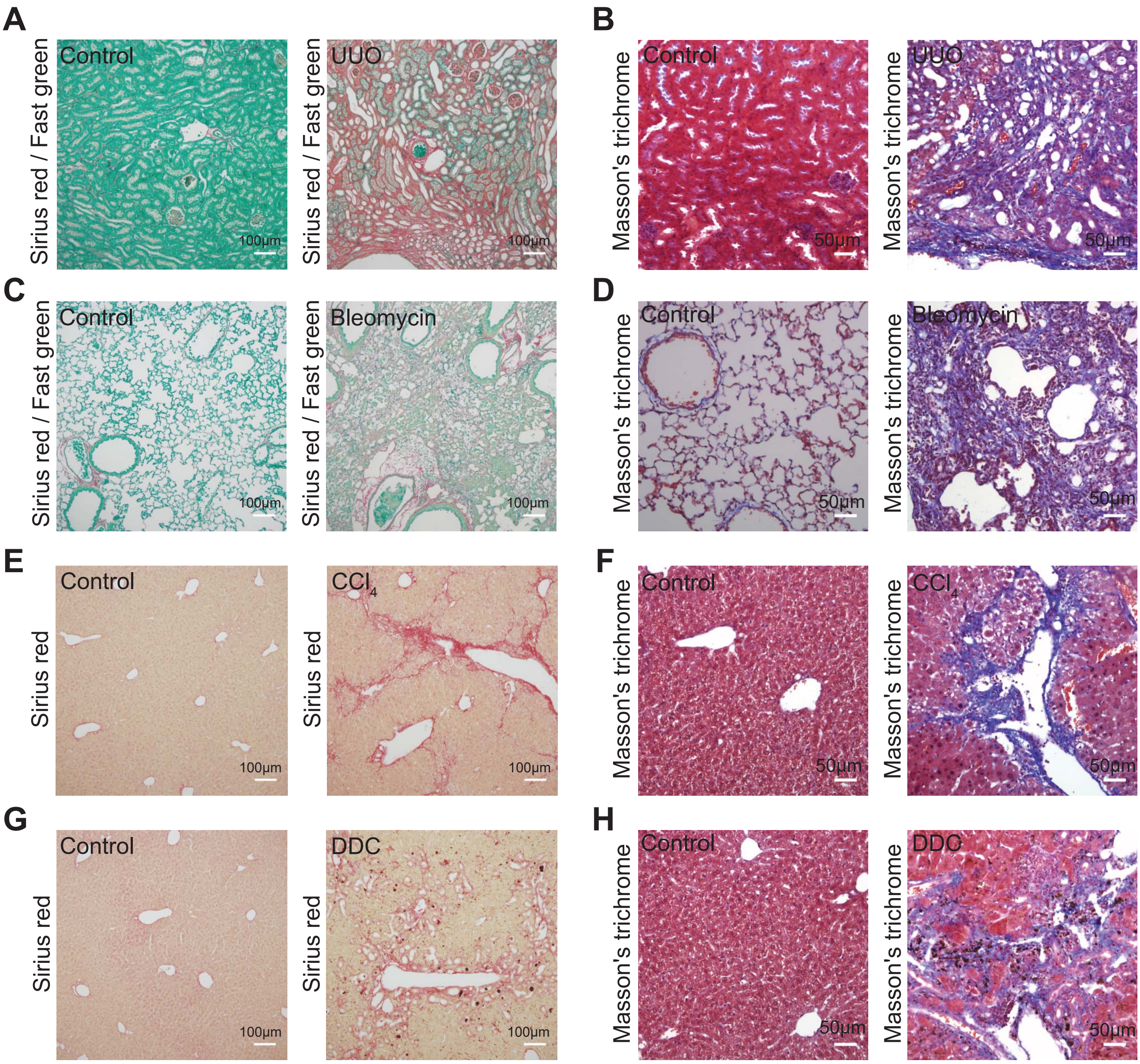
A
Sirius red / Fast green
Control
100µm
Sirius red / Fast green
UUO
100µm
B
Masson's trichrome
Control
50µm
Masson's trichrome
UUO
50µm
C
Sirius red / Fast green
Control
100µm
Sirius red / Fast green
Bleomycin
100µm
D
Masson's trichrome
Control
50µm
Masson's trichrome
Bleomycin
50µm
E
Sirius red
Control
100µm
Sirius red
CCl4
100µm
F
Masson's trichrome
Control
50µm
Masson's trichrome
CCl4
50µm
G
Sirius red
Control
100µm
Sirius red
DDC
100µm
H
Masson's trichrome
Control
50µm
Masson's trichrome
DDC
50µm

Figure S5

Figure S5. Histological staining showed the establishment of organ fibrosis.

(A) Sirius red and fast green staining of kidney sections from normal and UUO-induced mice, demonstrating collagen formation in renal fibrosis.

(B) Masson's trichrome staining of normal and fibrotic tissue sections demonstrating collagen formation in renal fibrosis.

(C) Sirius red and fast green staining of lung sections from normal and bleomycin-induced mice, demonstrating collagen formation in pulmonary fibrosis.

(D) Masson's trichrome staining of normal and fibrotic tissue sections demonstrating collagen formation in pulmonary fibrosis.

(E) Sirius red staining of liver sections from normal and CCl_4-induced mice, demonstrating collagen formation in liver fibrosis.

(F) Masson's trichrome staining of normal and fibrotic tissue sections demonstrating collagen formation in CCl_4-induced liver fibrosis.

(G) Sirius red staining of liver sections from normal and DDC-induced mice, demonstrating collagen formation in liver fibrosis.

(H) Masson's trichrome staining of normal and fibrotic tissue sections demonstrating collagen formation in DDC-induced liver fibrosis.

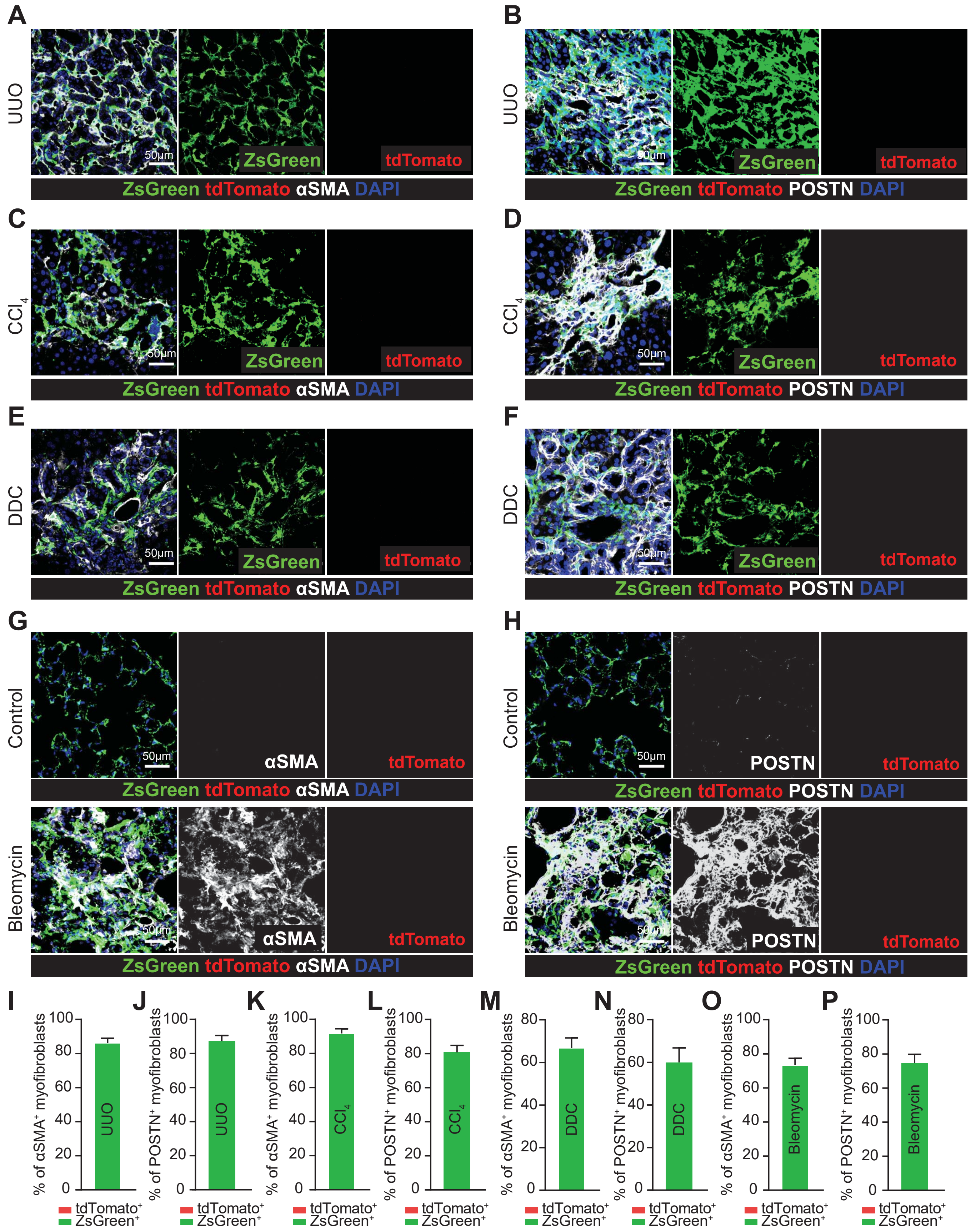

A
B
C
D
E
F
G
H
UUO
CCl4
DDC
Control
Bleomycin
ZsGreen
tdTomato
αSMA
POSTN
ZsGreen tdTomato αSMA DAPI
ZsGreen tdTomato POSTN DAPI
50µm
I
J
K
L
M
N
O
P
% of αSMA⁺ myofibroblasts
% of POSTN⁺ myofibroblasts
tdTomato⁺
ZsGreen⁺

Figure S6

Figure S6. Immunostaining showed the absence of BM-MSC-derived myofibroblasts in distal organ fibrosis.

(A-H) Confocal imaging of normal or fibrotic tissue sections revealed most αSMA^+ myofibroblasts and $POSTN^+$ myofibroblasts were $ZsGreen^+$ in renal fibrosis (A,B), liver fibrosis (C-F) and pulmonary fibrosis (G,H).

(I-P) Quantification of the percentages of αSMA^+ myofibroblasts and $POSTN^+$ myofibroblasts that were $ZsGreen^+$ or $tdTomato^+$ in renal fibrosis (I,J), liver fibrosis (K-N) and pulmonary fibrosis (O,P). $n = 5$ mice from 4 independent experiments.

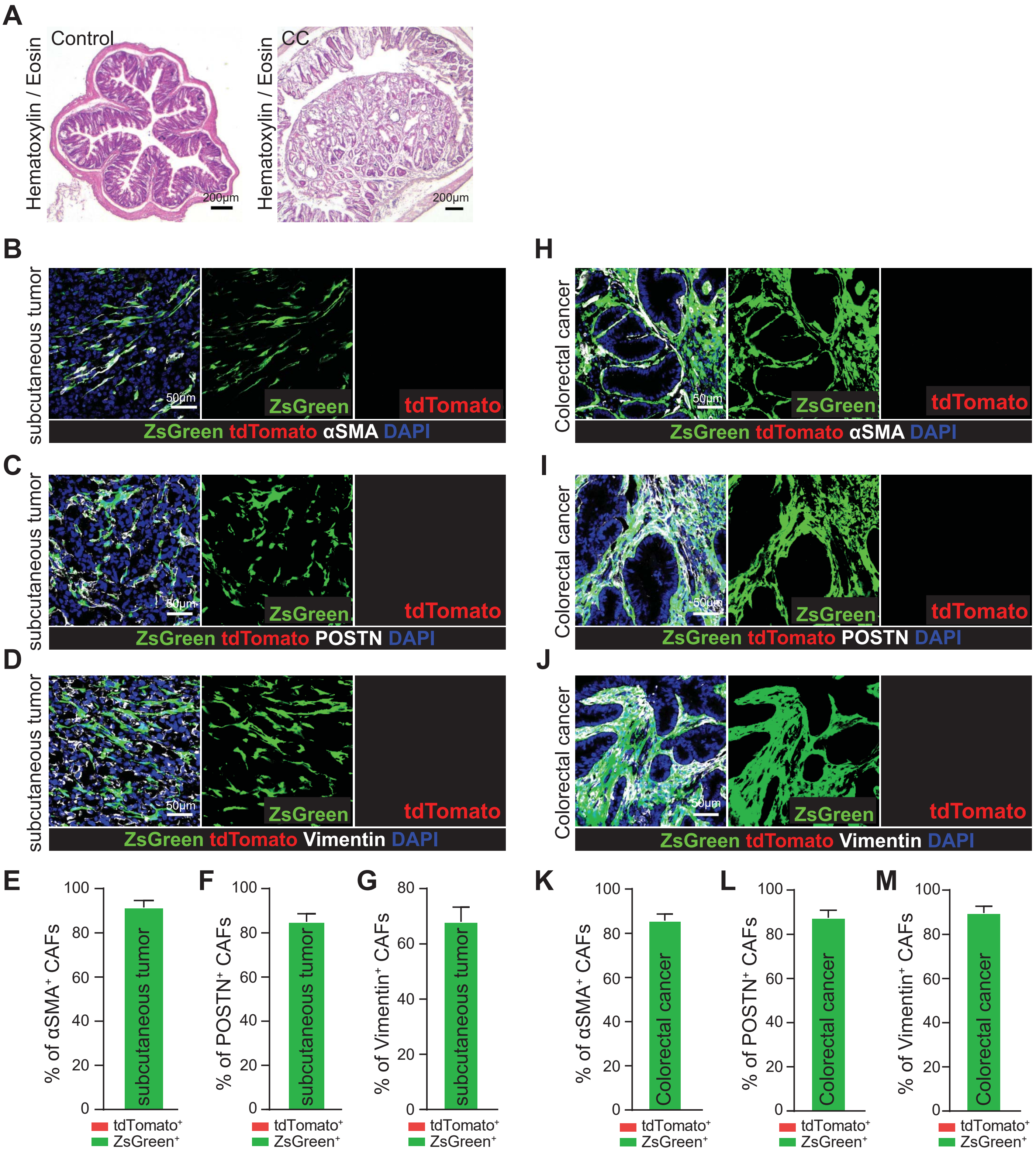
A
Hematoxylin / Eosin
Control
200μm
Hematoxylin / Eosin
CC
200μm
B
subcutaneous tumor
50μm
ZsGreen
tdTomato
ZsGreen tdTomato αSMA DAPI
C
subcutaneous tumor
50μm
ZsGreen
tdTomato
ZsGreen tdTomato POSTN DAPI
D
subcutaneous tumor
50μm
ZsGreen
tdTomato
ZsGreen tdTomato Vimentin DAPI
H
Colorectal cancer
50μm
ZsGreen
tdTomato
ZsGreen tdTomato αSMA DAPI
I
Colorectal cancer
50μm
ZsGreen
tdTomato
ZsGreen tdTomato POSTN DAPI
J
Colorectal cancer
50μm
ZsGreen
tdTomato
ZsGreen tdTomato Vimentin DAPI
E
% of αSMA+ CAFs
subcutaneous tumor
tdTomato+
ZsGreen+
F
% of POSTN+ CAFs
subcutaneous tumor
tdTomato+
ZsGreen+
G
% of Vimentin+ CAFs
subcutaneous tumor
tdTomato+
ZsGreen+
K
% of αSMA+ CAFs
Colorectal cancer
tdTomato+
ZsGreen+
L
% of POSTN+ CAFs
Colorectal cancer
tdTomato+
ZsGreen+
M
% of Vimentin+ CAFs
Colorectal cancer
tdTomato+
ZsGreen+

Figure S7

Figure S7. Immunostaining showed the absence of BM-MSC-derived CAFs in different tumor models.

(A) Hematoxylin and eosin (H&E) staining of colon sections from normal and AOM-DSS induced mice, demonstrating the development of colorectal cancer.

(B-D) Confocal imaging of subcutaneous tumors revealed most αSMA^+ CAFs (B), $POSTN^+$ CAFs (C) and $Vimentin^+$ CAFs (D) were $ZsGreen^+$.

(E-G) Quantification of the percentages of αSMA^+ CAFs (E), $POSTN^+$ CAFs (F) and $Vimentin^+$ CAFs (G) that were $ZsGreen^+$ or $tdTomato^+$ in E0771 implantation induced subcutaneous tumors. *n* = 3 mice from 3 independent experiments.

(H-J) Confocal imaging of colorectal cancer revealed most αSMA^+ CAFs (H), $POSTN^+$ CAFs (I) and $Vimentin^+$ CAFs (J) were $ZsGreen^+$.

(K-M) Quantification of the percentages of αSMA^+ CAFs (K), $POSTN^+$ CAFs (L) and $Vimentin^+$ CAFs (M) that were $ZsGreen^+$ or $tdTomato^+$ in AOM-DSS induced colorectal cancer. *n* = 3 mice from 3 independent experiments.

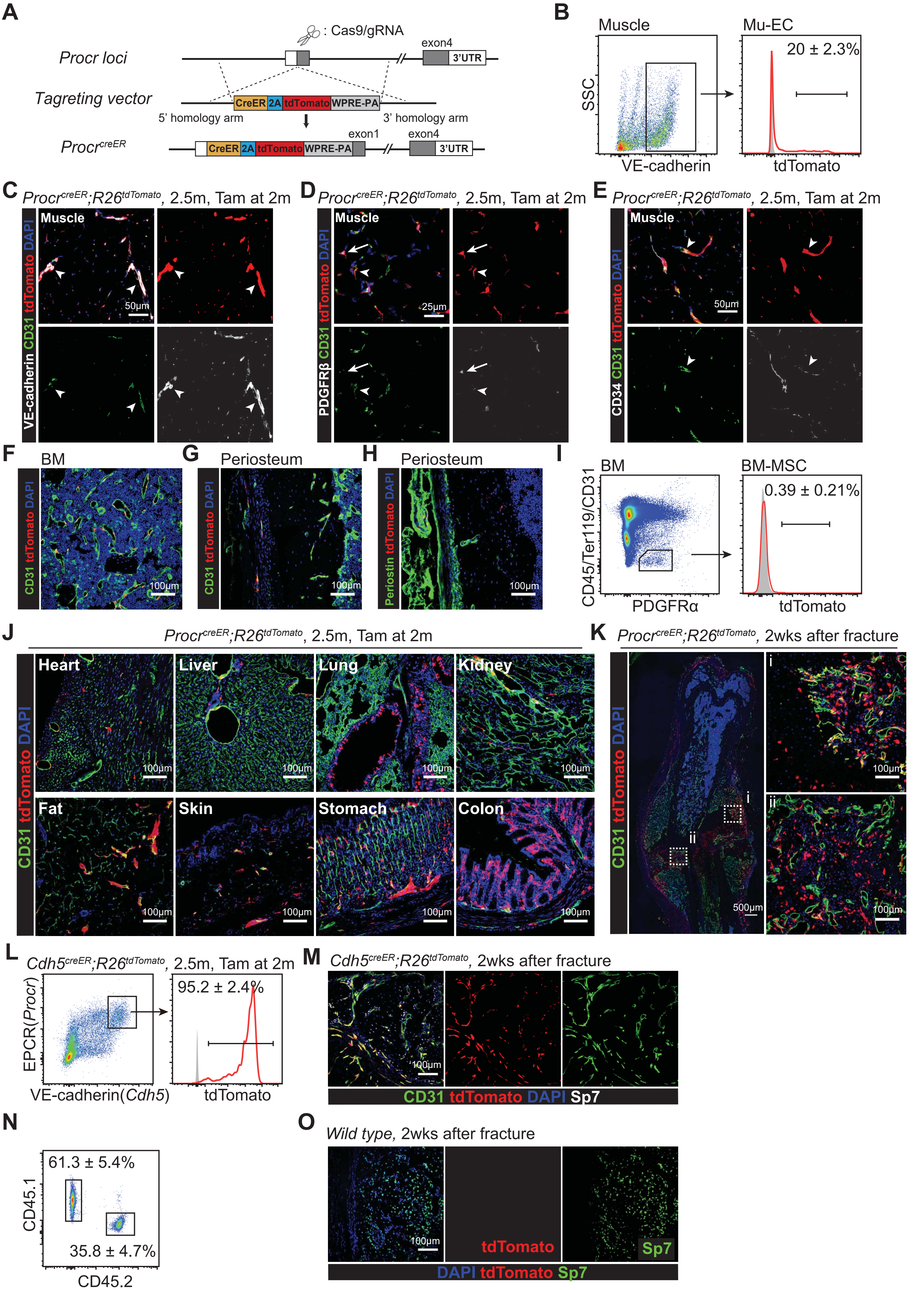
A
Procr loci
: Cas9/gRNA
exon4
3'UTR
Tagreting vector
CreER 2A tdTomato WPRE-PA
5' homology arm
3' homology arm
Procr^creER
exon1
B
Muscle
Mu-EC
20 ± 2.3%
SSC
VE-cadherin
tdTomato
C
Procr^creER;R26^tdTomato, 2.5m, Tam at 2m
Muscle
VE-cadherin CD31 tdTomato DAPI
50µm
D
PDGFRβ CD31 tdTomato DAPI
25µm
E
CD34 CD31 tdTomato DAPI
F
BM
CD31 tdTomato DAPI
100µm
G
Periosteum
H
Periostin tdTomato DAPI
I
BM-MSC
0.39 ± 0.21%
CD45/Ter119/CD31
PDGFRα
J
Heart
Liver
Lung
Kidney
Fat
Skin
Stomach
Colon
K
Procr^creER;R26^tdTomato, 2wks after fracture
500µm
i
ii
L
Cdh5^creER;R26^tdTomato, 2.5m, Tam at 2m
95.2 ± 2.4%
EPCR(Procr)
VE-cadherin(Cdh5)
M
Cdh5^creER;R26^tdTomato, 2wks after fracture
CD31 tdTomato DAPI Sp7
N
61.3 ± 5.4%
35.8 ± 4.7%
CD45.1
CD45.2
O
Wild type, 2wks after fracture
tdTomato
Sp7
DAPI tdTomato Sp7

Figure S8

Figure S8. *Procr*-CreER traced MSCs in muscle but not in BM.

(A) Schematic illustration of the design and generation of *Procr*^{creER} mice.

(B) Flow cytometric analysis of enzymatically digested muscle cells showed the percentages of VE-cadherin$^+$ endothelial cells (Mu-EC) that were tdTomato$^+$. n = 4 mice from 4 independent experiments.

(C) Confocal imaging of muscle sections from 2.5-month-old *Procr*^{creER};*R26*^{tdTomato} mice at 2 weeks postinduction revealed all tdTomato$^+$CD31$^+$ endothelial cells expressed VE-cadherin.

(D,E) Confocal imaging of muscle sections from 2.5-month-old *Procr*^{creER};*R26*^{tdTomato} mice at 2 weeks postinduction revealed tdTomato$^+$CD31$^+$ endothelial cells did not express the MSC markers PDGFRβ (D) or CD34 (E).

(F,G) Confocal imaging of bone marrow (BM) and periosteum showed CD31$^+$ endothelial cells in BM (F) and periosteum (G) were labeled by tdTomato in 2.5-month-old *Procr*^{creER};*R26*^{tdTomato} mice treated with tamoxifen at 2 months of age.

(H) Confocal imaging of periosteum showed Periostin$^+$ periosteal stromal cells were tdTomato$^-$ in 2.5-month-old *Procr*^{creER};*R26*^{tdTomato} mice treated with tamoxifen at 2 months of age.

(I) Flow cytometric analysis of enzymatically digested BM cells showed the percentages of CD45$^-$Ter119$^-$CD31$^-$PDGFRα$^+$ BM-MSCs (BM-MSC) that were tdTomato$^+$. n = 3 mice from 3 independent experiments.

(J) Confocal imaging of frozen sections from different organs showed CD31$^+$ endothelial cells were tdTomato$^+$ in 2.5-month-old *Procr*^{creER};*R26*^{tdTomato} mice treated with tamoxifen at 2 months of age.

(K) Confocal imaging of femur sections from *Procr*^{creER};*R26*^{tdTomato} mice at 2 weeks after femur fracture showed some CD31$^+$ endothelial cells in the callus were marked by tdTomato.

(L) Flow cytometric analysis of enzymatically dissociated muscle from 2.5-month-old *Cdh5*^{creER};*R26*^{tdTomato} mice at 2 weeks postinduction revealed efficient labeling of EPCR$^+$VE-cadherin$^+$ endothelial cells by tdTomato. n = 3 mice from 3 independent experiments.

(M) Confocal imaging of femur sections from *Cdh5*^{creER}*;R26*^{tdTomato} mice at 2 weeks after femur fracture showed only CD31^{+} endothelial cells in the callus were marked by tdTomato.

(N) Flow cytometry analysis of peripheral blood from wild-type parabionts after parabiosis between *Procr*^{creER}*;R26*^{tdTomato} mice (CD45.2) and wild-type mice (CD45.1). *n* = 3 mice from 3 independent experiments.

(O) Confocal imaging of femurs from wild-type parabionts two weeks after fracture.

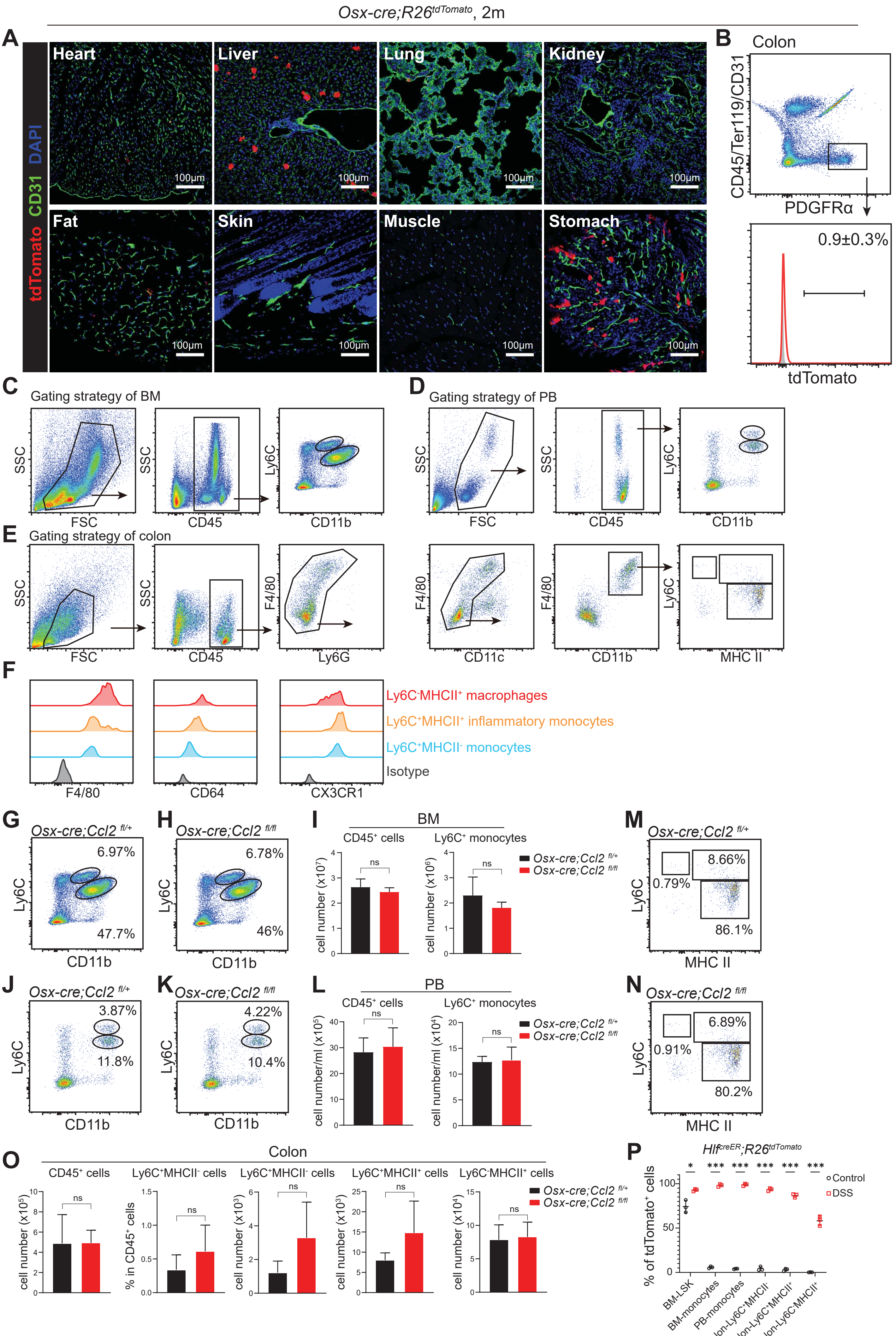

Osx-cre;R26tdTomato, 2m
A
tdTomato CD31 DAPI
Heart
Liver
Lung
Kidney
Fat
Skin
Muscle
Stomach
100µm
B
Colon
CD45/Ter119/CD31
PDGFRα
0.9±0.3%
tdTomato
C
Gating strategy of BM
SSC
FSC
CD45
Ly6C
CD11b
D
Gating strategy of PB
E
Gating strategy of colon
Ly6G
F4/80
CD11c
MHC II
F
Ly6C-MHCII+ macrophages
Ly6C+MHCII+ inflammatory monocytes
Ly6C+MHCII- monocytes
Isotype
F4/80
CD64
CX3CR1
G
Osx-cre;Ccl2 fl/+
6.97%
47.7%
H
Osx-cre;Ccl2 fl/fl
6.78%
46%
I
BM
CD45+ cells
Ly6C+ monocytes
cell number (x10^7)
cell number (x10^6)
ns
Osx-cre;Ccl2 fl/+
Osx-cre;Ccl2 fl/fl
M
Osx-cre;Ccl2 fl/+
8.66%
0.79%
86.1%
J
Osx-cre;Ccl2 fl/+
3.87%
11.8%
K
Osx-cre;Ccl2 fl/fl
4.22%
10.4%
L
PB
CD45+ cells
Ly6C+ monocytes
cell number/ml (x10^5)
cell number/ml (x10^4)
N
Osx-cre;Ccl2 fl/fl
6.89%
0.91%
80.2%
O
Colon
CD45+ cells
Ly6C+MHCII- cells
Ly6C+MHCII- cells
Ly6C+MHCII+ cells
Ly6C-MHCII+ cells
cell number (x10^5)
% in CD45+ cells
cell number (x10^3)
cell number (x10^3)
cell number (x10^4)
P
HlfcreER;R26tdTomato
% of tdTomato+ cells
Control
DSS
*
***
BM-LSK
BM-monocytes
PB-monocytes
Colon-Ly6C+MHCII-
Colon-Ly6C+MHCII+
Colon-Ly6C-MHCII+

Figure S9

Figure S9. Ccl2 deletion in BM-MSCs did not affect the frequencies of immune cells under normal conditions.

(A) Confocal imaging of frozen sections from different organs of 2-month-old *Osx-cre;R26*^{tdTomato} mice showed perivascular stromal cells were tdTomato$^-$. Endothelial cells were indicated with anti-CD31 antibody staining.

(B) Flow cytometric analysis of enzymatically digested colon cells from 2-month-old *Osx-cre;R26*^{tdTomato} mice showed CD45$^-$Ter119$^-$CD31$^-$PDGFRα$^+$ colon stromal cells were tdTomato$^-$.

(C-E) Flow cytometric gating strategy for identifying monocytes in the bone marrow (BM; C), peripheral blood (PB; D), and colon (E).

(F) Flow cytometric analysis of F4/80, CD64, and CX3CR1 expression in Ly6C$^+$MHCII$^-$ monocytes, Ly6C$^+$MHCII$^+$ inflammatory monocytes, and Ly6C$^-$MHCII$^+$ macrophages.

(G-I) Flow cytometric analysis showed the percentages of Ly6C$^+$ monocytes in bone marrow (BM) from control (G) and *Osx-cre;Ccl2*$^{fl/fl}$ mice (H) under normal conditions. The numbers of CD45$^+$ leukocytes and Ly6C$^+$ monocytes in BM were quantified (I).

(J-L) Flow cytometric analysis showed the percentages of Ly6C$^+$ monocytes in peripheral blood (PB) from control (J) and *Osx-cre;Ccl2*$^{fl/fl}$ mice (K) under normal conditions. The numbers of CD45$^+$ leukocytes and Ly6C$^+$ monocytes in PB were quantified (L).

(M-O) Flow cytometric analysis showed the percentages of Ly6C$^+$MHCII$^-$ monocytes, Ly6C$^+$MHCII$^+$ inflammatory monocytes and Ly6C$^-$MHCII$^+$ macrophages in colon from control (M) and *Osx-cre;Ccl2*$^{fl/fl}$ mice (N) under normal conditions. The numbers of CD45$^+$ leukocytes, Ly6C$^+$MHCII$^-$ monocytes, Ly6C$^+$MHCII$^+$ inflammatory monocytes and Ly6C$^-$MHCII$^+$ macrophages in colon were quantified (O). All data represent mean ± SD from 5 mice across 3 independent experiments. Statistical difference was measured by two-tailed Student's *t*-tests (ns: not significant).

(P) Flow cytometric analysis of BM, PB, and colon from 2.5-month-old *Hlf*^{creER}*;R26*^{tdTomato} mice showing tdTomato$^+$lineage$^-$c-Kit$^+$Sca-1$^+$ hematopoietic stem and progenitor cells (HSPCs) gave rise to monocytes and macrophages in the colon after DSS treatment. All data represent mean ± SD from 3 mice across 3 independent

experiments. Statistical difference was measured by two-tailed Student's *t*-tests (*$p < 0.05$, ***$p < 0.001$).

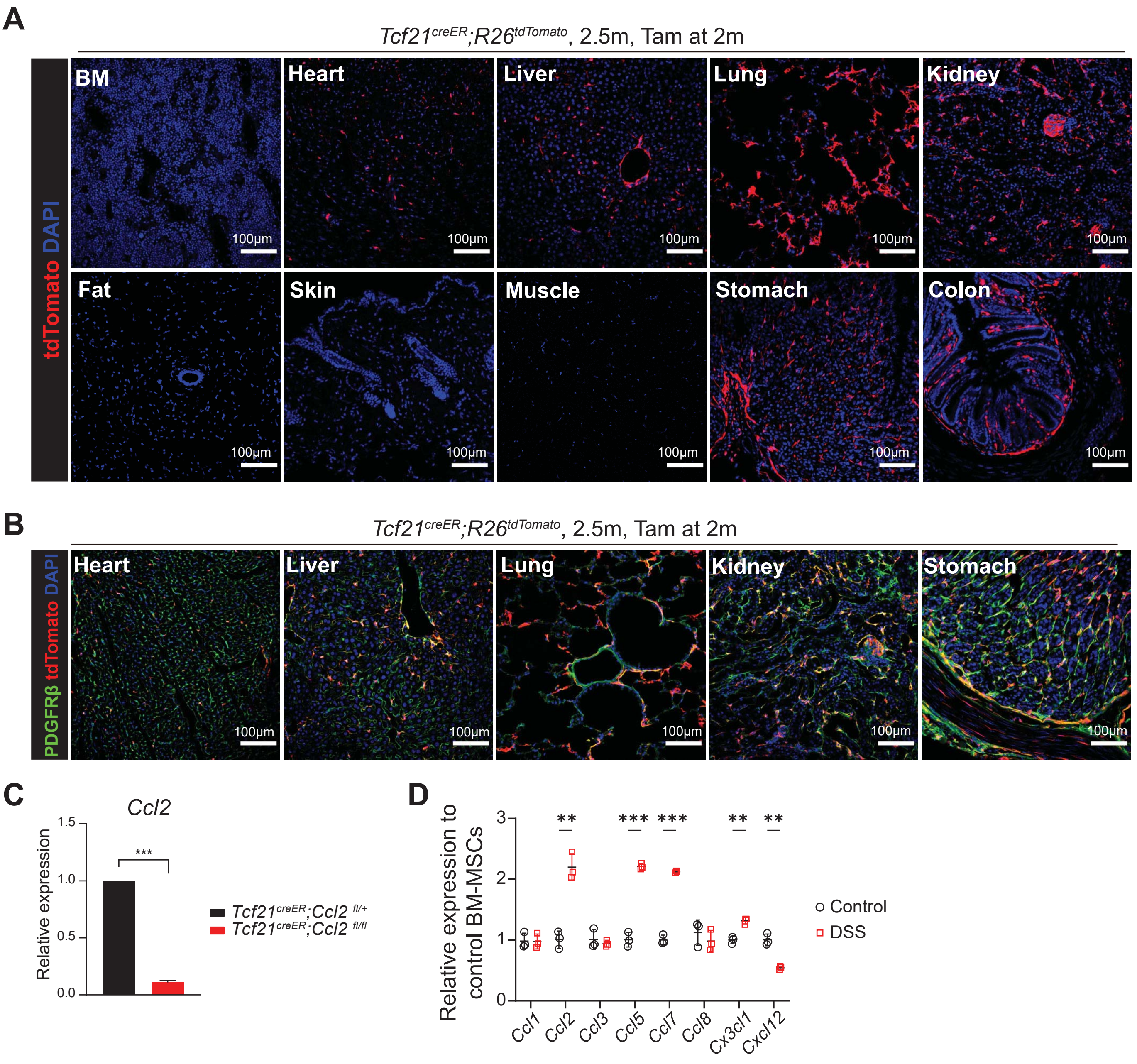

A
Tcf21creER;R26tdTomato, 2.5m, Tam at 2m
tdTomato DAPI
BM
Heart
Liver
Lung
Kidney
Fat
Skin
Muscle
Stomach
Colon
100μm
B
Tcf21creER;R26tdTomato, 2.5m, Tam at 2m
PDGFRβ tdTomato DAPI
Heart
Liver
Lung
Kidney
Stomach
100μm
C
Ccl2
Relative expression
Tcf21creER;Ccl2 fl/+
Tcf21creER;Ccl2 fl/fl
***
D
Relative expression to control BM-MSCs
Control
DSS
Ccl1
Ccl2
Ccl3
Ccl5
Ccl7
Ccl8
Cx3cl1
Cxcl12

Figure S10

Figure S10. *Tcf21*-CreER enabled specific labeling of MSCs in multiple internal organs.

(A) Confocal imaging demonstrated that tdTomato$^+$ cells were selectively present in heart, liver, lung, kidney, stomach and colon.

(B) Confocal imaging revealed efficient labeling of PDGFRβ$^+$ stromal cells by tdTomato in different internal organs of 2.5-month-old *Tcf21^{creER};R26^{tdTomato}* mice treated with tamoxifen at 2 months old.

(C) Quantitative real-time PCR analyses of the transcript levels (normalized to *Gapdh*) of *Ccl2* in *Tcf21^{creER};Ccl2$^{fl/fl}$* mice and their controls. All data represent mean ± SD from 3-5 mice. Two-tailed Student's *t* tests were used to assess the statistical significance of differences between sex-matched littermates (***p < 0.001).

(D) Relative mRNA expression levels of *Ccl1*, *Ccl2*, *Ccl3*, *Ccl5*, *Ccl7*, *Ccl8*, *Cx3cl1* and *Cxcl12* in CD45$^-$Ter119$^-$CD31$^-$PDGFRα$^+$ BM-MSCs isolated from control or DSS-treated wild-type mice. All data represent mean ± SD from 3 mice. Two-tailed Student's *t* tests were used to assess the statistical significance of differences between sex-matched littermates (**p < 0.01, ***p < 0.001).
